# Comparative metabolic profiling of olive leaf extracts from twelve different cultivars collected in both fruiting and flowering seasons

**DOI:** 10.1038/s41598-022-27119-5

**Published:** 2023-01-12

**Authors:** Eman M. Kabbash, Zeinab T. Abdel-Shakour, Sherweit H. El-Ahmady, Michael Wink, Iriny M. Ayoub

**Affiliations:** 1 Phytochemistry Department, National Organization for Drug Control and Research, Giza, Egypt; 2 Department of Pharmacognosy, Faculty of Pharmacy, Ain Shams University, Cairo, 11566 Egypt; 3 Institute of Pharmacy and Molecular Biotechnology, Heidelberg University, INF 364, 69120 Heidelberg, Germany

**Keywords:** Secondary metabolism, Plant sciences

## Abstract

*Olea europaea* is an economically significant crop native to Mediterranean countries. Its leaves exhibit several biological properties associated to their chemical composition. The aqueous ethanolic extracts of olive leaves from twelve different cultivars were analyzed by high performance liquid chromatography coupled to photodiode array and electrospray ionization mass spectrometry (HPLC/PDA/ESI–MS/MS). A total of 49 phytochemicals were identified in both positive and negative ionization modes. The identified compounds belonged to four classes of secondary metabolites including secoiridoids, flavonoids, pentacyclic triterpenoids and various phenolic compounds. Seasonal variation in chemical composition among the studied cultivars was apparent in autumn and spring. Secologanoside, oleuropein, hydroxy-oleuropein, demethyl oleuropein, gallocatechin, luteolin-*O*-hexoside, diosmetin, oleanolic acid and maslinic acid were detected in all cultivars in both seasons. Oleuropein-*O*-deoxyhexoside was tentatively identified for the first time in olive leaf extracts; detected only in the Spanish cultivar Picual (PIC) collected in spring. Also, dihydroxy-oxooleanenoic acid and hydroxy-oxooleanenoic acid, two bioactive pentacyclic triterpenes, were identified. Principle component analysis (PCA) showed good discrimination among the studied cultivars in terms of their botanical origin. This study is considered the first study for non-targeted metabolic profiling of different olive leaf cultivars cultivated in Egypt.

## Introduction

Traditional Mediterranean diet is associated with low incidence of vascular and heart diseases in addition to certain cancer types^1^. These health benefits could be attributed to the diversity of the Mediterranean diet which is rich in various antioxidants that are important in disease prevention^2^.

Olive tree (*Olea europaea* L.) is native to Mediterranean countries. Olive fruits are considered as an economically significant crop and its oil, rich in unsaturated fatty acids has apparent health benefits^3^. Moreover, olive leaf extracts have been recently marketed as dietary product^4^. Commercial products in the form of herbal teas or food supplements are available all over the world, as complete dried leaves, powder, extracts or tablets^5^. The leaves have characteristic profiles of phytochemicals^6^. They are considered as potential source for various classes of bioactive compounds such as secoiridoids, flavonoids, phenolic acids, coumarins, and triterpenes^7^. Among these several constituents, oleuropein and its hydrolysis derivatives were the main biophenol secoiridoids found in olive tree which are known for their beneficial properties for health. Besides, the amount and nature of flavonoids in the olive leaves have also an important impact on their biological properties. In this context, numerous flavonoid aglycones (luteolin, diosmetin, quercetin, apigenin) were found in olive together with flavonoid glycosides such as luteolin-*O*-rutinoside, luteolin-*O*-glucoside, quercetin-*O*-rutinoside^8^.

The phenolic profile of olive leaves can vary significantly based on the variety, the geographical origin, as well as the sampling time^9,10^. Plant metabolomics could help to elucidate the complexity of phytochemicals present. Recently, development in plant metabolomics techniques, allows the detection of several hundred metabolites simultaneously and comparing samples reliably to identify differences and similarities in an untargeted manner^11^. Several analytical techniques have been developed for then on-targeted profiling of metabolites in plants. These include proton nuclear magnetic resonance (^1^H-NMR)^12^, high performance liquid chromatography–mass spectrometry (HPLC–MS)^13,14^, gas chromatography-mass spectrometry (GC–MS)^15^, and direct injection Fourier-transform ion cyclotron resonance mass spectrometry (FTICR-MS)^16^.

Separations based mass spectrometry approaches, such as LC–MS and GC–MS, are highly sensitive and provide excellent identifying capacity. A study was conducted on Spanish olive leaf extracts using HPLC coupled to electrospray time-of-flight mass spectrometry (ESI-TOF–MS) and electrospray ion trap multiple-stage tandem mass spectrometry (ESI-IT-MS^*n*^) for the screening of phenolic compounds in the leaf extracts^17^. In another study HPLC–MS was used for phenolic profiling of olive bark and leaves^18^, and HPLC–DAD/ESI–MS/MS was employed to determine the phenolic constituents in Tunisian cultivars extra virgin olive oil and the effect of adding olive leaves on the oil composition^19^. Recently, several studies focused on combining various analytical techniques with multivariate analysis, which became of exciting potential for the plant metabolomics field^20^. Such an approach was used recently to investigate the effect of genotypes, climate, season variation, and extract processing, including the drying conditions, temperature, light, and oxygen exposure in phenolic profiles of olive leaves. Among these, a study was performed on nine different olive leaf extracts showed significant variation on phenolic concentrations based on genotypes using GGE biplot analysis^21^. Another study carried on 15 olive leaf varieties using high-performance liquid chromatography coupled with electrospray ionization and quadrupole time-of flight mass spectrometry showed that the types and concentrations of phenolic substances greatly influenced by variety type depending on Pearson’s and Spearman’s rank correlation analyses along with PCA^10^. Besides, a more comprehensive study on 32 olive leaves cultivars grown in China was performed using PCA as one of multivariate data analysis techniques. This study revealed the discrimination of cultivars based upon their phytochemical profiles and antioxidant capacities^22^.

Quality assessment of the leaf extracts of twelve olive cultivars was carried out earlier to emphasize the impact of seasonal variation on oleuropein content, total flavonoid and total polyphenol content via HPLC and UV spectroscopy coupled to multivariate data analyses^23^. In the current study, metabolites fingerprints of olive leaves were acquired using liquid chromatography coupled to mass spectrometry and successively analyzed to provide a more comprehensive overview on the phytochemical profile of olive leaves. The processed data were subjected to multivariate analysis using PCA to highlight compositional differences among twelve different olive cultivars cultivated in Egypt and to predict the effect of seasonal variation on the leaf extracts collected in fruiting and flowering seasons. Additionally, LC–MS metabolic fingerprinting of each extract combined with PCA analysis was used in untargeted manner for genotype classification and identification of the most important secondary metabolites responsible for such classification. To the best of our knowledge, this metabolomics-based comparative approach provides the first comprehensive study on the differences between olive leaf different cultivars grown in Egypt.

## Results and discussion

### HPLC–PDA-ESI–MS/MS analysis

**Figure 1:**
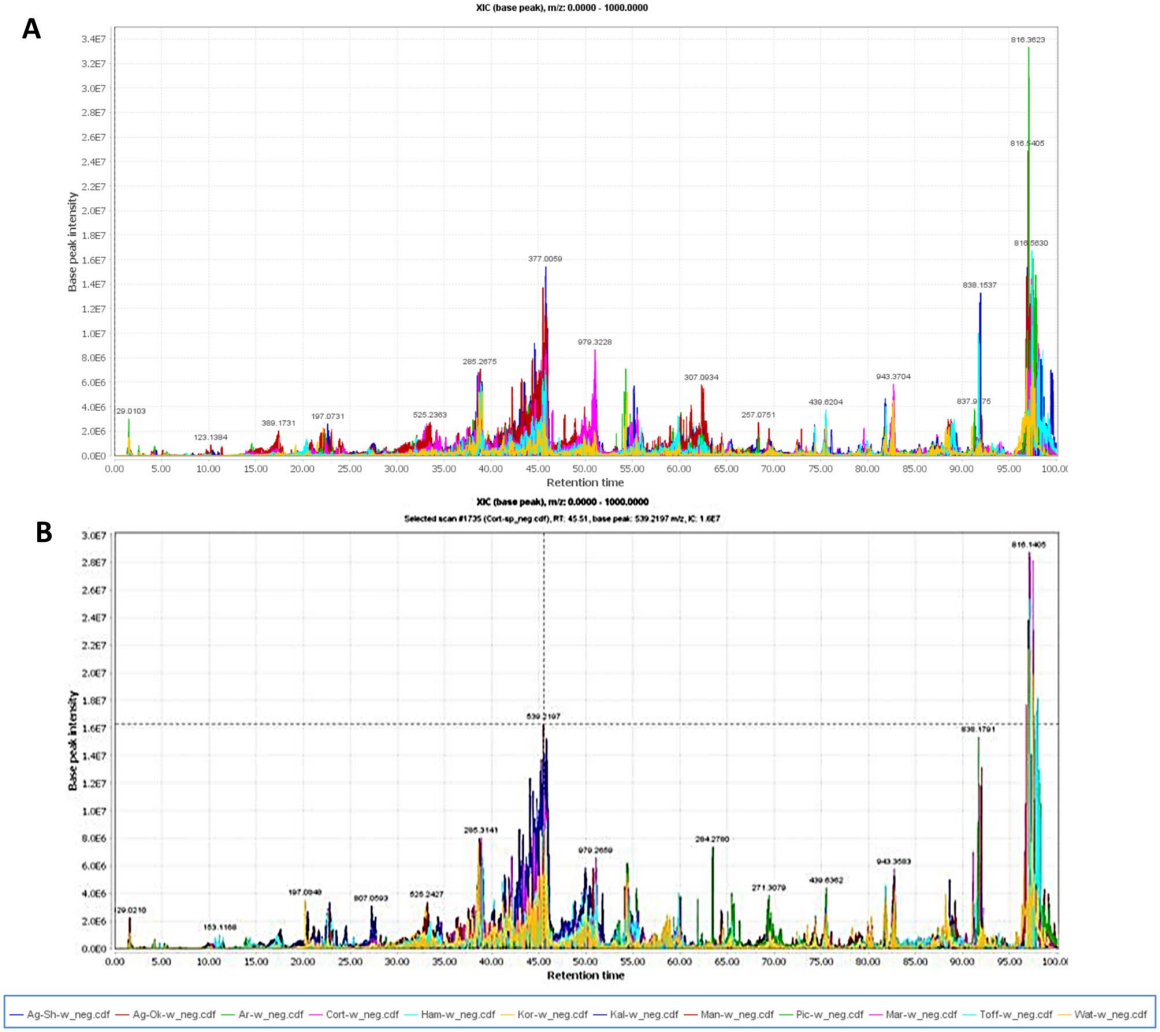
Total ion chromatogram of olive leaves from twelve cultivars collected in autumn (**A**) and spring (**B**), in the negative ionization mode.

**Figure 2:**
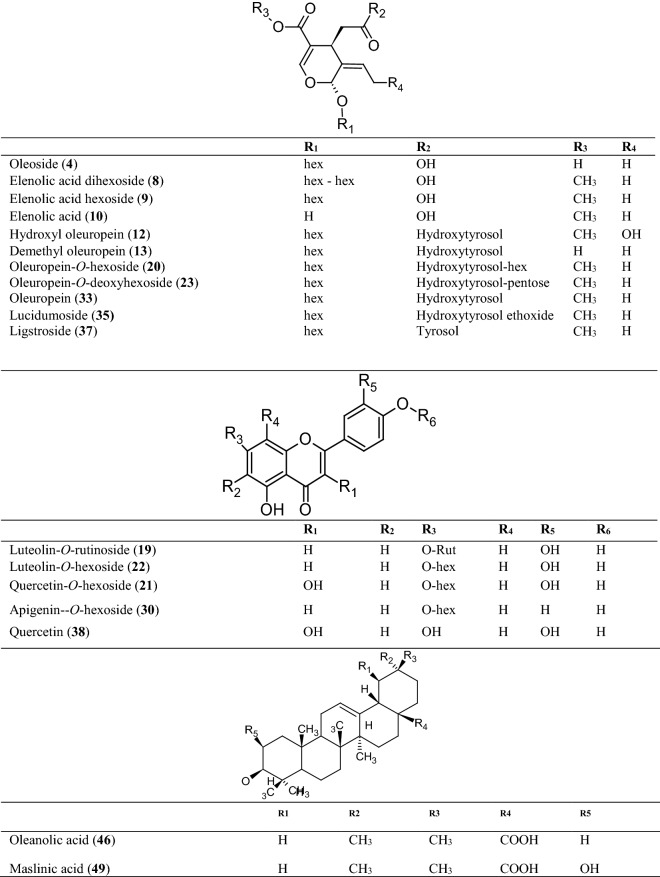
Representative classes of main metabolites tentatively identified in *O. europaea* leaf extracts: (**A**) secoiridoids, (**B**) flavonoids, (**C**) pentacyclic triterpenes with selected compounds discussed in the manuscript.

The chemical composition of olive leaf extracts from twelve cultivars collected in autumn and spring were analyzed using HPLC coupled to ion trap mass spectrometer with an ESI source. All extracts were analyzed in both positive and negative modes to cover compounds with diverse ionization responses. ESI^−^ has been previously used to determine the structure of flavonoid glycosides^24,25^, iridoids, and triterpenes; whereas, coumarins, and alkaloids showed better ionization in ESI^+^ positive mode^26,27^. A comprehensive metabolite profiling was performed for all olive leaf extracts. A total of 49 metabolites were annotated belonging to 4 different classes including secoiridoids, flavonoids, triterpenoids, and various other phenolic compounds. Table 1 shows the compounds that were tentatively identified in *O. europaea* leaf extracts. The elution order is based mainly on their polarity, the more polar the compound the shorter the retention time. The total ion chromatogram of the twelve cultivars collected in autumn (**A**) and spring (**B**), in the negative ionization mode is presented in Fig. 1. The base peak chromatograms of individual olive leaf extracts analyzed in negative ionization mode in both seasons are displayed in supplementary figure (Fig. S1). Structures of selected metabolites identified in *O. europaea* leave extracts belonging to secoiridoids (A), flavonoids (B) and pentacyclic triterpenes (C) were illustrated in Fig. 2.

**Table 1:** Metabolites identified in *O. europaea* leaf extracts by LC/MS in both positive and negative modes.

Peak no	R_*t*_ (min)	UV (nm)	Molecular formula	[M−H]^−^	[M+H]^+^	MS^n^ ions (*m/z*)	Metabolite	Class	References
1	1.61	233, 326	C_7_H_11_O_6_^−^	191.27		173,127,111, 93, 85	Quinic acid	Organic acid	^59^ ^60^
2	8.12	250	C_16_H_23_O_10_^−^	374.98		331, 213, 169, 151	Loganic acid	Iridoid	^36^
3	10.15	220, 283	C_8_H_9_O_3_^−^	153.02		123	Hydroxytyrosol	Simple phenol	^33^
4	10.46	270	C_16_H_21_O_11_^−^	389.00		345, 227, 209, 183, 165	Oleoside	Secoiridoid	^32^
5	14.69	270	C_16_H_21_O_11_^−^	389.00		345, 227, 209, 183	Secologanoside	Secoiridoid	^32^
6	17.72	277	C_9_H_9_O_5_^−^	196.90		169, 151, 125	Ethyl gallate	Phenolic acid derivative	^42^
7	23.91	275	C_15_H_13_O_7_^−^	305.13		245, 225, 97	(Epi) Gallocatechin	Flavan-3-ol	^61^
8	23.97	232, 284	C_23_H_33_O_16_^−^	565.09		533,403,241	Elenolic acid dihexoside	Secoiridoid glycoside	^62^
9	26.07	234, 272	C_17_H_23_O_11_^−^	403.08		371, 241, 223, 179	Elenolic acid hexoside/oleoside methyl ester	Secoiridoid glycoside	^33^
10	31.37	240, 269	C_11_H_13_O_6_^−^	240.95	243	209, 165, 139, 121	Elenolic acid	Secoiridoid	^33^
11	32.8	279, 379	C_14_H_5_O_8_^−^	301.08		257,241, 229, 185	Ellagic acid	Phenolic acid	^42^
12	33.02	224, 282	C_25_H_31_O_14_^−^	555.22		537, 393, 323, 291	Hydroxy-oleuropein	Secoiridoid	^32^
13	33.29	271	C_24_H_29_O_13_^−^	525.30		363, 319, 249	Demethyl-oleuropein	Secoiridoids	^17^
14	33.99	245, 283	C_31_H_41_O_17_^−^	685.16		523, 453, 421, 385	(Iso)Nuezhenide	Iridoid glycoside	^36^
15	34.85	280, 360	C_27_H_29_O_17_^−^	625.15		463, 301	Quercetin-di-*O*-hexoside	Flavonol glycoside	Massbank
16	34.95	269, 365	C_27_H_29_O_16_^−^	609.27		447, 301, 179	Quercetin-*O*-hexoside-*O*-deoxyhexoside	Flavonol glycoside	Massbank
17	36.21	269, 370	C_27_H_29_O_16_^−^	609.20		463, 447, 301, 271, 179	Quercetin-*O*-deoxyhexosylhexoside	Flavonol glycoside	^39^
18	36.86	260, 345	C_27_H_29_O_15_^−^	593.26		447, 431, 285	Luteolin-*O*-robinoside	Flavone glycoside	^61^
19	37.02	260, 345	C_27_H_29_O_15_^−^	593.14		447, 285	Luteolin-*O*-rutinoside	Flavone glycoside	^32^
20	38.2	246, 283	C_31_H_41_O_18_^−^	701.18		565, 539, 377, 307, 275	Oleuropein-*O*-hexoside	Secoiridoid	^63^
21	38.43	265, 370	C_21_H_19_O_12_^−^	463.06		301, 271,179	Quercetin-*O*-hexoside	Flavonol glycoside	^38^
22	38.44	249, 345	C_21_H_19_O_11_^−^	447.11	449	327,285, 199, 179, 151	Luteolin-*O*-hexoside	Flavone glycoside	^17^
23	39.41	255, 280	C_31_H_41_O_17_^−^	685.04		539, 377, 307, 275	Oleuropein-*O*-deoxyhexoside	Secoiridoid	–
24	39.48	255, 360	C_23_H_21_O_13_^−^	505.30		463, 301	Unknown	Flavonoid	–
25	41.38	260, 340	C_33_H_39_O_18_^−^	723.30		577, 559, 457, 269	Apigenin-*O*-dideoxyhexoside-hexoside	Flavone glycoside	–
26	41.52	260, 340	C_27_H_29_O_14_^−^	577.13	579.01	415, 269	Apigenin-*O*-hexosyldeoxyhexoside	Flavone glycoside	Pubchem
27	42	249, 284, 330	C_29_H_36_O_15_^−^	623.32		461,342,315	Verbascoside	Phenylethanoid glycoside	^33^
28	42.69	235, 345	C_28_H_31_O_15_^−^	607.22		299, 284	Diosmin	Flavone glycoside	^61^
29	43.12	260, 337	C_27_H_31_O_14_^+^		579.21	433, 417, 271	Apigenin-*O*-deoxyhexoside-*O*-glucoside	Flavone glycoside	^61^
30	43.58	260, 337	C_21_H_19_O_10_^−^	431.31	433.02	269	Apigenin-*O*-hexoside	Flavone glycoside	^38^
31	43.96	246	C_25_H_29_O_15_^−^	569.04		537, 407, 389	Oleuropeinic acid	Secoiridoid	^37^
32	45.14	235, 345	C_22_H_21_O_11_^−^	461.01	462.98	299, 285	Diosmetin-*O*-hexoside	Flavone glycoside	^38^
33	45.49	230, 281	C_25_H_31_O_13_^−^	539.14		377, 307, 275, 223	Oleuropein	Secoiridoid	^35^
34	45.47	225, 280	C_25_H_33_O_13_^−^	541.12		378, 308, 276	Hydro oleuropein	Secoiridoid	^61^
35	46.53	230, 280	C_27_H_35_O_14_^−^	583.09		537, 461, 375, 273	Lucidumoside C	Secoiridoid glycoside	^36,60^
36	49.91	nd	C_20_H_23_O_6_^+^		359	341,327,235, 219, 205	Pinoresinol	Lignan	^38^ , FooDB
37	50.84	235, 280	C_25_H_32_O_12_^−^	523.17		361, 291, 259, 223	Ligstroside	Secoiridoid glycoside	^17^
38	55.50	265, 370	C_15_H_11_O_7_^+^		303	285, 257, 165, 137	Quercetin	Flavonol	HMDB
39	55.91	268, 350	C_15_H_9_O_6_^−^	285.23		257, 151, 133, 107	Luteolin	Flavone	^61^
40	61.11	230, 280	C_42_H_54_O_23_^−^	925.04		893, 539, 377, 345, 307	Jaspolyoside	Secoiridoid glycoside	^61^
41	62.76	236, 282	C_19_H_21_O_8_^−^	377.17		345, 307, 275, 241	Oleuropein aglycone	Secoiridoid	^33^
42	64.86	267, 337	C_16_H_11_O_6_^−^	299.03	301	285, 151	Diosmetin	Flavone	^38^
43	74.30	235	C_30_H_49_O_5_^+^		489.01	471, 425	Dihydroxyursolic acid	Triterpenoid	^36^ FooDB
44	75.61	234	C_30_H_47_O_5_^+^		487.36	469, 439, 405	Dihydroxy-oxo-oleanenoic acid	Triterpenoid	^64^
45	76.68	234	C_30_H_47_O_4_^+^		471.14	453, 425, 407, 395	Hydroxy-oxo-oleanenoic acid		
46	79.75	235	C_30_H_49_O_3_^+^		457.25	439, 411, 393, 191	Oleanolic acid/Ursolic acid	Triterpenoid	^65^
47	83.8	234	C_30_H_51_O_2_^+^		443.15	425, 407, 289	Uvaol/eryrthodiol	Triterpenoid	HMDB
48	84.29	234	C_30_H_49_O_3_^+^		457.22	439, 393, 176	Betulinic acid	Triterpenoid	
49	86.47	234	C_30_H_49_O_4_^+^		473.35	455, 427, 409	Maslinic acid	Triterpenoid	^47^, HMDB

#### Secoiridoids

*Oleaeuropaea* is rich in secoiridoids; especially the esterified forms with a phenolic moiety are known as oleosides^28^. Lack of characteristic chromophores for most of secoiridoids renders UV spectra usually of limited use^29^. However, secoiridoids are characterized by marked absorption bands at λ_max_ 240 nm and 270 nm^30^. HPLC–MS fragmentation pathways were used to determine the molecular formula and the loss of characteristic moieties. The fragment ions appeared are corresponding to distinctive losses, such as [M−H−CH_3_OH]^−^, [M−H−CH_3_OH−H_2_O]^−^, [M−H−C_4_H_6_O]^−^, and a characteristic fragment usually appears due to McLafferty rearrangement for the phenyl ester fragment^31^.

##### Oleoside derivatives

Compounds (**4**) and (**5**) eluted at R_*t*_ 10.04 and 14.69 min, respectively, showed a molecular ion peak [M−H]^−^ at *m/z* 389 related to oleoside and its isomer secologanoside. Their MS^2^ spectra (Supp. Figs. S2, S3) exhibited a fragment ion peak at *m/z* 345 arising from the loss of CO_2_ (44 Da) of a carboxylic group while the product ion at *m/z* 277 was related to the loss of a hexose moiety (162 Da). A fragment ion peak at *m/z* 183 indicated a subsequent loss of CO_2_. This fragmentation pattern emphasizes the presence of two carboxylic groups and a hexose moiety. Secologanoside is eluted after oleoside in reversed phase conditions and exhibits a strong peak at *m/z* 345^32^.

##### Elenolic acid derivatives

Compound (**9**) eluted at R_*t*_ 26.07 min; showed a deprotonated molecular ion peak [M−H]^−^ at *m/z* 403. Its second order spectrum led to the formation of product ions at *m/z* 371 arising from the loss of CH_3_OH [M−H−CH_3_OH]^−^, *m/z* 241 corresponding to the loss of a hexose moiety [M−H−162]^−^, and a fragment ion at *m/z* 223 related to the formation of dehydrated elenolic acid by subsequent loss of a H_2_O molecule. This fragmentation pattern is consistent with that reported for elenolic acid hexoside; a degradation product of oleuropein^33^. The molecular ion peak [M−H]^−^ of compound (**8**) (*m/z* 565.09) was 162 Da more than compound (**9**), indicating an additional hexose moiety. Thus compound (**8**) was identified as elenolic acid dihexoside. HPLC–MS data for compound (**10**), eluted at R_*t*_31.37 min, showed a molecular ion peak [M−H]^−^ at *m/z* 240.95. MS^2^ spectrum (Supp. Fig. S4) revealed fragment ions at *m/z* 209, 165, 139, and 121 corresponding to the loss of CH_3_OH [M−H-32]^−^, followed by a subsequent loss of CO_2_ [M−H-32-44]^−^. A base peak appeared at *m/z* 139 corresponding to the characteristic cleavage in the iridoid ring [M−C_4_H_6_O]^−^. This fragmentation pattern was consistent with the data reported for elenolic acid^33^. Elenolic acid is considered as a degradation product of oleuropein and is used as a marker for olive maturation^34^. It is worth mentioning that elenolic acid could not be detected in all leaf extracts during spring.

##### Oleuropein derivatives

Nine known oleuropein derivatives were identified, exhibiting similar UV absorption maxima and mass fragmentation pattern^35^. They all showed fragment peaks corresponding to characteristic losses of hexose moiety [M−H-162]^−^, followed by subsequent loss of C_4_H_6_O [M−H-162-70]^−^, and CH_3_OH moieties [M−H−162-70-32]^−^.

A major peak at R_*t*_ 45.49 min was obvious in all the olive leaf extracts. It exhibited a molecular ion peak [M−H]^−^ at *m/z* 539.14. MS^2^ fragmentation revealed a base peak at *m/z* 377 corresponding to the characteristic loss of a hexose moiety [M−H−162]^−^, two additional peaks at *m/z* 307 and *m/z* 275 corresponding to subsequent loss of C_4_H_6_O [M−H−162-70]^−^, and CH_3_OH groups [M−H−162–70-32]^−^, respectively. This is consistent with the fragmentation pattern of oleuropein (**33**) (Supp. Fig. S5). Oleuropein is an ester of hydroxytyrosol with *β*-glucosylated elenolic acid.

Compounds (**12**, **13**, **20**, **34**, **35**, **37and 41**) eluted at R_*t*_ (33.02, 33.29, 38.20, 45.47, 46.53, 51.74 and 62.76), respectively, were tentatively identified as oleuropein derivatives. They all showed fragment ion peaks corresponding to characteristic losses of hexose moiety [M−H-162]^−^, followed by subsequent losses of C_4_H_6_O [M−H-162-70]^−^, and CH_3_OH groups [M−H-162-70-32]^−^.

Compound (**12**) showed a precursor ion at *m/z* 555.22, with 16 Da higher than oleuropein, indicating a hydroxy oleuropein derivative and exhibiting the same fragmentation pattern of oleuropein at (*m/z* 393, 323, 291)^32^. Compound (**13**) showed a molecular ion peak [M−H]^−^ at *m/z* 525.30 relative to demethyl oleuropein. Its MS^2^ spectrum showed fragment ions at (*m/z* 363, 293, 261). Compound (**20**) has a molecular ion peak at *m/z* 701.23, with 162 Da higher than oleuropein, indicating the presence of one excess hexose moiety. Its MS^2^ showed a characteristic peak due to the loss of a hexose moiety producing daughter ions relative to oleuropein and its fragments at (*m/z* 539, 377, 307, 275). Compound (**20**) was identified as oleuropein-*O*-hexoside**.** Compound (**34**) showed a molecular ion peak at m/z 541.12; 2 Da higher than oleuropein and its fragment ions at (*m/z* 378, 308, 276); thus, it was identified as hydro oleuropein.

Lucidumoside C (**35**); a secoiridoid glycoside was previously isolated from *Ligustrum lucidum*^36^. It has a structure similar to oleuropein with additional ethoxide moiety (*m/z* 583.09). MS^2^ spectrum showed a fragment ion peak at *m/z* 537 relative to the loss of an ethanol moiety [M−H-46]^−^, followed by similar fragmentation pattern to oleuropein (*m/z* 375, 305, 273).

Compound (**37**) was identified as ligstroside; a deoxy analogue of oleuropein; with a molecular ion peak at *m/z* 523.17 and fragment ion peaks at (*m/z* 361, 291, 259)^17^. Compound (**31**) eluting at R_*t*_ 43.96 min showed a molecular ion peak [M−H]^−^ at *m/z* 569.04 with a marked fragment ion at *m/z* 407 relative to the loss of hexose moiety [M−H-162]^−^, and another fragment ion at *m/z* 537 corresponding to [M−H−CH_3_OH]^−^; suggesting this molecule to be oleuropeinic acid^37^.

Compound (**23**) was traced only in PIC leaf extract in spring. It showed a molecular ion peak [M−H]^−^ at *m/z* 685.04. MS^2^ spectrum indicated a base peak at *m/z* 539 corresponding to oleuropein, marking the loss of deoxyhexose moiety [M−H-146]^−^ and fragment ion peaks at *m/z* 377, 307 and 275 which is the typical fragmentation pattern of oleuropein. Thus, compound (**23**) was tentatively identified as oleuropein-*O*-deoxyhexoside. To the best of our knowledge, this is the first report of oleuropein-*O*-deoxyhexoside in nature. The chemical structure and ESI–MS/MS spectrum of oleuropein-*O*-deoxyhexoside is illustrated in Supp. Fig. S6.

#### Flavonoids

Various flavonoids were previously reported in olive leaf extract, either in aglycone or glycosylated forms. In this work flavonoid identifications were based on studying their UV spectra and the mass spectrum of each identified compound.

##### Flavones

The UV/Vis spectra of flavones characterized by a λ_max_ for band Ι around 340 nm, as well it provides valuable information about the degree of hydroxylation as the increase in the number of hydroxyl groups increased λ_max_^38^. Their MS spectra used to determine molecular formula and identify the structure of the aglycone for the eluted flavonoid based on its fragmentation pattern.

Compound (**39**) showed a molecular ion peak at *m/z* 285.23 identified as luteolin based on the fragmentation pattern proposed for flavones. Its MS^2^ spectrum showed fragment ions at *m/z* 257 relative to loss of carbonyl group [M−H-28]^−^, *m/z* 151, 133 and 179 corresponding to ^1,3^A, ^1,3^B and ^0,4^B respectively (Supp. Fig. S7). Compounds (**18** and **19**) showed the same molecular ion peak at *m/z* 593.26, with similar fragmentation pattern. They showed a base peak at *m/z* 285 corresponding to luteolin aglycone. Fragment ions appeared at *m/z* 447 [M−H-146]^−^ and 285 [M−H-308]^−^ indicates the loss of deoxyhexose followed by hexose moiety (308 Da). Compound (**18**) was tentatively identified as luteolin-*O*-robinoside eluting before its isomer luteolin-*O*-rutinoside (**19**); that was previously isolated from olive leaf extracts^39^. A peak at R_*t*_ 38.62 min was obvious in all the examined cultivars. It showed a UV absorption maximum at 345 nm for band I characteristic for flavones. The EIC at *m/z* 447.11 with its strong fragment at *m/z* 285, due to the loss of a hexose residue, indicates luteolin-*O*-hexoside (**22**). Luteolin-7-*O*-glucoside was previously detected as a major compound in olive leaf extracts as well as in the fruits^17^. In parallel, the MS^2^ spectra of compounds **25**, **26, 29** and **30** exhibited a fragment ion peak at *m/z* 269 corresponding to apigenin moiety. Compound (**25**) showed a molecular ion peak [M−H]^−^ at *m/z* 723.3 similar to apigenin-*O*-dideoxyhexoside-hexoside. This was confirmed by examining its MS^2^spectrum, where characteristic ion peaks were observed at *m/z* 577 and 559, indicating a subsequent loss of deoxyhexose [M−H-146]^−^ followed by a water molecule [M−H-146-18]^−^. A base peak at *m/z* 269 was observed corresponding to apigenin aglycone. Compound (**26**) showed a molecular ion peak [M−H]^−^ at *m/z* 577.13 relative to apigenin-*O*-hexosyl deoxyhexoside. Its MS^2^ showed fragment ions at *m/z* 415 and 269 corresponding to the loss of hexose [M−H-162]^−^ followed by a rhamnose moiety [M−H-162-146]^−^. Compound (**29**) showed a molecular ion peak [M+H]^+^ at *m/z* 579.21 with fragment ions at *m/z* 433 relative to [M+H-146]^+^, *m/z* 417 [M+H-162] ^+^ and *m/z* 271 corresponding to apigenin aglycone. Compound **29** was identified as apigenin-*O*-deoxyhexoside-*O*-hexoside. Compound (**30**) was identified as apigenin-*O*-hexoside based on its molecular ion [M−H]^−^ at *m/z* 431.31; and fragment ion at *m/z* 269 relative to the loss of hexose moiety.

Among the identified flavones: several diosmetin derivatives were tentatively assigned in olives leaves. Compound (**42**) showed a molecular ion peak [M−H]^−^ at *m/z* 299.10 and its fragment at *m/z* 285. Thus compound (**42**) was tentatively identified as diosmetin. While compound (**28**) showed a molecular ion peak [M−H]^−^ at *m/z* 607.22; 308 Da higher than diosmetin corresponding to rutinoside moiety. It was identified as diosmin based on its fragmentation pattern. Compound (**32**) showed a molecular ion peak [M+H]^+^ at *m/z* 462.98 with a base peak at *m/z* 301 corresponding to the loss of hexose moiety [M+H-162]^+^, thus compound (**32**) was identified as diosmetin-*O*-hexoside^38^.

##### Flavanols

The UV data of compounds **15**, **17**, **21, 24** and **38** showed two absorption bands at the range of 240–280 nm and 330–380 nm; suggesting these peaks are flavonol derivatives. They all exhibited characteristic fragment ion peak at *m/z* 301 corresponding to quercetin aglycone resulting from the subsequent losses of pentose, hexose and acetyl hexose sugars (− 132, − 162, and − 204 Da, respectively).

Compound (**15**) displayed a deprotonated molecular ion [M−H]^−^at *m/z* 625.15 with fragment ions at *m/z* 463 and 301 relative to the successive loss of two hexose moieties indicating quercetin di-*O*-hexoside. Compound (**17**) displayed a molecular ion peak [M−H]^−^ at *m/z* 609.20. The MS^2^ spectrum displayed a fragment ion peak at *m/z* 301, indicating the elimination of a rutinosyl residue (308 Da). Furthermore, product ions at *m/z* 463 and 447 corresponding to loss of deoxyhexose and hexose moieties indicate the elution of quercetin-*O*-deoxyhexoside-*O*-hexoside^39,40^. The MS spectra of peak (**21**) showed a molecular ion peak [M−H]^−^ at *m/z* 463.06; the MS^2^ spectrum for this ion displayed fragment ion peaks at *m/z* 301, 271 and 179.The base peak appeared at *m/z* 301 corresponding to the loss of a hexose moiety and the mass of the remaining aglycone part (quercetin).Also, the appearance of two fragment ion peaks at *m/z* 271 [M−H–CO−H]^−^ and *m/z* 179 corresponds to the cleavage in ring C, thus confirms the structure of quercetin-*O*-hexoside. Compound (**38**) displayed a molecular ion [M+H]^+^ peak at *m/z* 303 and fragment ions at *m/z* 165 and 137 corresponding to ^0,2^A and ^0,2^B fragments arise from c ring cleavage. Furthermore, product ions at *m/z* 257 and 229 corresponding to [M+H–COOH]^+^ and [M+H–CO_2_–CO–H]^+^ are characteristic to quercetin.

#### Additional phenolics and phenolic acids

Compound (**3**) showed a UV spectrum with two λ_max_ at 220 and 283 nm. It exhibited a molecular ion peak [M-H]^−^ at *m/z* 153 and a fragment ion peak at *m/z*123 corresponding to the loss of a CH_2_OH group. This was consistent with hydroxytyrosol, one of the main components of olive leaves, as previously described^41^. Peak **6**showed a λ_max_ near 280 nm. Its mass spectrum showed a molecular ion peak [M−H]^−^ at *m/z* 197.Its MS^2^revealed fragment ions at *m/z* 169 corresponds to the loss of C_2_H_4_ moiety [M−H-28]^−^ and *m/z* 123due to subsequent loss of H_2_O molecule^42^. Thus, compound **6** was identified as ethyl gallate; an ethyl ester of gallic acid. It was previously identified in Chinese olive^43^.

Peak (**11**) showed a UV spectrum with λ_max_ 276and 376 nm. It displayed a deprotonated molecular ion [M−H]^−^ at *m/z* 301. Its MS^2^ spectrum showed three characteristic fragments at *m/z* 257, 229, 185 due to subsequent losses of CO_2_ (44 Da), followed by the loss of CO (28 Da) and another molecule of CO_2_, thus, this compound was tentatively identified as ellagic acid^42^. Compound (**27**) showed a molecular ion peak [M−H]^−^ at *m/z* 623.25 with its product ions at *m/z* 461 due to loss of caffeic acid moiety, weak ion at *m/z* 315 due to loss of rhamnose unit and a fragment ion at *m/z* 161 due to proton transfer of the remaining ketene fragment. This is coincident with that reported for verbascoside fragmentation pattern^33^. Thus, compound 27 was identified as verbascoside;a heterosidic ester of caffeic acid and hydroxytyrosol which was previously detected in appreciable amount in mature olive leaves^44^.

#### Pentacyclic triterpenoids

*O. europaea* fruit and leaf have been reported as a rich source of triterpenic acids and pentacyclic triterpenols either free or esterified with fatty acids^45^. Among these, oleanolic, ursolic, maslinic acids are the most prominent triterpene acids in the olive leaves, as well as, uvaol, erythrodiol as triterpene alcohols^46^.

A common fragmentation pattern for all pentacyclic triterpenes priveously isolated from olive leaves is the dehydration step [M−H_2_O]^+^. Peak **47** showed a molecular ion peak at *m/z* 457 relative to the molecular formula of C_30_H_48_O_3_**.** Its fragmentation pattern showed peaks at *m/z* 439, 393 and 191 corresponding to the mass spectrum of oleanolic acid charachterized by the loss of H_2_O moiety [M+H-18]^+^, followed by CO_2_ [M+H-44]^+^ and the last fragment is charachtaristic to pentacyclic triterpenoids corresponding to [M+H-C_15_H_23_]^−^ moiety.

Although most interests are directed toward the phenolic composition of olive leaf extract; olive leaves have been reported as a rich source of bioactive pentacyclic triterpenes^45^. Triterpenes are characterized by diverse pharmacological properties including hepatoprotective, anti-inflammatory, antimicrobial, anti-hyperlipidemic, gastro protective and antidiabetic effects^47^. Guinda et al. 2010b determined the triterpenoid content of the fruits and leaves of three Spanish cultivars. Results showed that the levels of triterpenes in the leaf extract were 30 fold higher than those found in the fruit^45^.

Herein, the triterpenoid contents studied for different cultivars were shown to be dependent on the variety, and in all cases oleanolic acid was the major triterpenic compound. Hydroxy-oxooleanenoic acid (**45**) and dihydroxy-oxooleanenoic acid (**44**), are first identified in olive leaf extracts according to our knowledge. They were identified in all the studied cultivars. Hydroxy-oxooleanenoic acid and its derivatives were shown to possess antimicrobial and cytotoxic activities against wide tumor cell lines^48^. Thus, olive leaf extract might serve as a source for such valuable anti-tumor agent.

### Multivariate data analysis of HPLC–MS data

**Figure 3:**
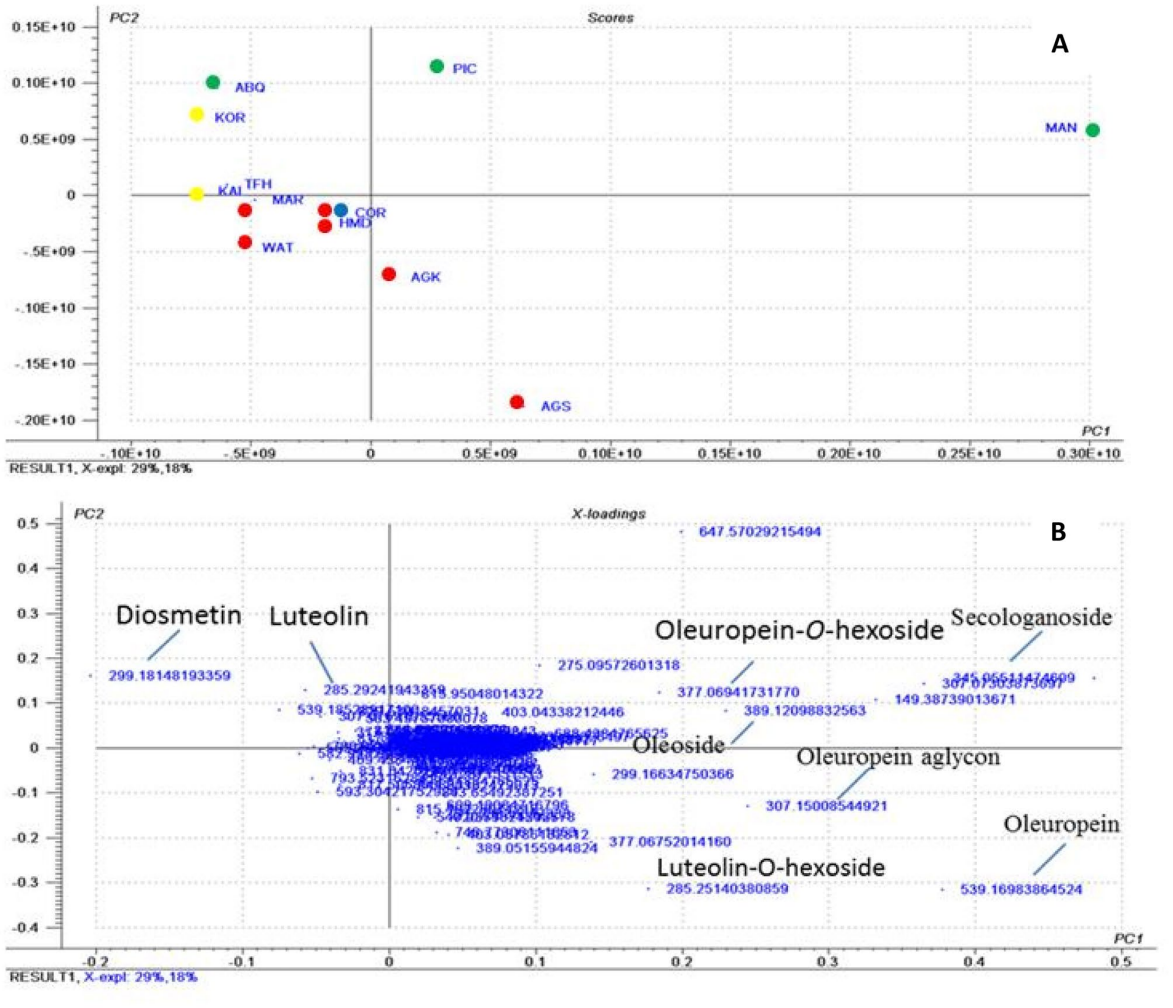
PCA analysis of different cultivars of olive leaves collected in autumn derived from the negative ionization mode HPLC/ MS data (*m/z* 100–1000); showing (**A**) a PCA score plot of metabolites of olive leaves and (**B**) a loading plot of metabolites of olive leaves. *AOK* Agizi Okasi, *ASH* Agizi Shami, *HMD* Hamed, *MRK* Maraki, *TFH* Toffahi, *WAT* Watiken, *MAN* Manzanillo, *PIC* Picual, *ABQ* Arbequina, *KAL* Kalamata, *KOR* Koroneiki, *COR* Coratina. Where, Egyptian cultivars in red, Spanish cultivars in green, Greek cultivars in blue and Italian cultivars in yellow.

PCA as unsupervised multivariate data analysis technique was performed to explain metabolite differences and possible discrimination between the studied cultivars in an untargeted manner. The aligned peak lists obtained from the processing of HPLC–MS data of the negative ionization mode for autumn and spring extracts were subjected to PCA analysis. The score plot obtained for autumn extracts (Fig. 3A) showed two orthogonal PCs, accounting for 47% of the variance among the data. The score plot showed marked segregation among cultivars in relation to their botanical origin, where the three Spanish cultivars located positive to PC_2_, while the Egyptian cultivars are clustered in the negative side. The two Greek cultivars (KAL and KOR) are grouped together in the upper left quadrant. The PCA was able to differentiate between Egyptian cultivars and others that reveal differences in their composition based on their botanical origin. By examining the loading plot (Fig. 3B) to explain the underlying reasons for such clustering; flavonoids and secoiridoids were found to contribute the most in species discrimination. They segregate the cultivars into two groups one positive to PC_1_ for cultivars rich in secoiridoids including MAN, PIC, AOK and ASH. Another group clustered negative to PC_2_ includes (ABQ, KOR, KAL, COR, TFH, MRK, HMD and WAT) related to their high flavonoids. Concerning to the identified metabolites, diosmetin was found to be more enriched in KOR, KAL, ABQ and TFH. On the other hand, the two Spanish cultivars MAN and PIC were found to be rich in secologanoside, oleoside and oleuropein-*O*-hexoside; whereas oleuropein, oleuropein aglycone and luteolin-*O*-hexoside were found to be more enriched in the Egyptian cultivars ASH and AOK.

**Figure 4:**
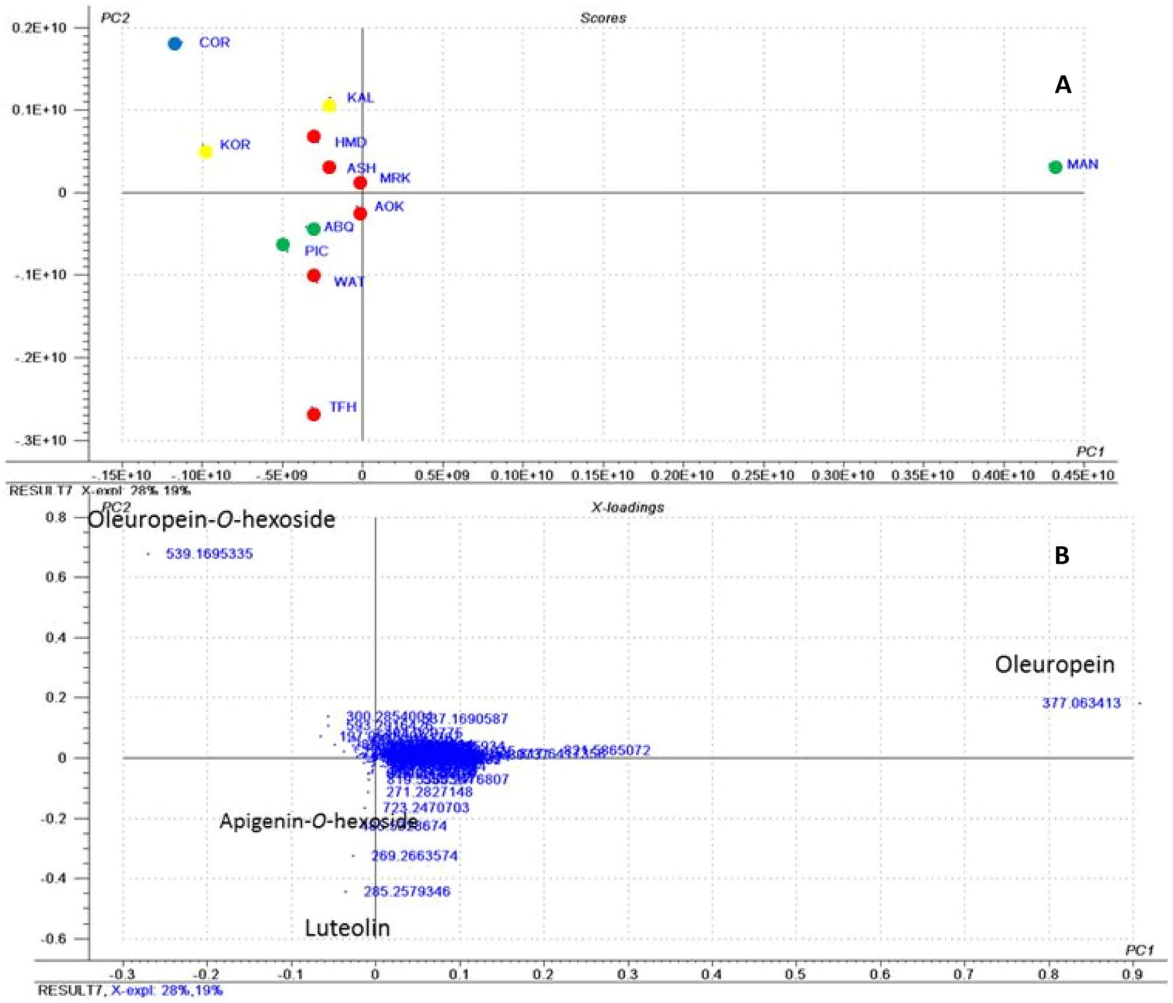
PCA analysis of different cultivars of olive leaves collected in spring derived from the negative ionization mode HPLC/MS data (m/z 100–1000); showing (**A**) a PCA score plot of metabolites of olive leaves and (**B**) a loading plot of metabolites of olive leaves. *AOK* Agizi Okasi, *ASH* Agizi Shami, *HMD* Hamed, *MRK* Maraki, *TFH* Toffahi, *WAT* Watiken, *MAN* Manzanillo, *PIC* Picual, *ABQ* Arbequina, *KAL* Kalamata, *KOR* Koroneiki, *COR* Coratina. Where, Egyptian cultivars in red, Spanish cultivars in green, Greek cultivars in blue and Italian cultivars in yellow.

PCA analysis for spring extracts was performed. The obtained score and loading plots (Fig. 4A,B), showed the segregation of cultivars based on their metabolites. Here again flavonoids and secoiridoids were found to contribute the most in species discrimination. Negative ion-mode MS, in which metabolites are deprotonated has the potential to increase the coverage of phenolic compounds analysis^49^. In the present study, most of phenolics are more likely to retain a negative charge and thus large amount of data generated in the negative mode. Therefore, negative ionization data has been chosen for principal component analysis (PCA). Two groups were observed; one positive to PC_2_ includes MAN, COR, KAL, KOR, HMD, ASH and MRK. The second group clustered negative to PC_2_ includes AOK, ABQ, PIC, WAT and TFH. In terms of the identified metabolites oleuropein and oleuropein-*O*-hexoside were found to be more abundant in the first group, while the second group was found to be richer in apigenin-*O*-hexoside and luteolin.

### Seasonal variation in olive leaf composition

By examining the TIC for all extracts in both seasons in the phenolic region, it was obvious that spring is characterized by higher oleuropein content for most of the studied cultivars. This can be correlated with the absence of elenolic acid; a degradation product of oleuropein; in all leaf extracts during spring. Certain compounds were identified in all the studied olive cultivars leaf extract. They were present in all cultivars in both seasons but differ quantitatively. They include secologanoside (**5**), gallocatechin (**7**), elenolic acid hexoside (**9**), hydroxy-oleuropein (**12**), demethyl oleuropein (**13**), luteolin–*O*-rutinoside (**19**), oleuropein-*O*-glucoside (**20**), luteolin-*O*-hexoside (**22**), apigenin-*O*-hexoside (**30**), oleuropein (**33**), jaspolyoside (**40**), oleuropein aglycone (**41**), diosmetin (**42**), dihydroxy-oxo-oleanenoic acid (**44**), hydroxy-oxo-oleanenoic acid (**45**), oleanolic acid (**46**), and maslinic acid (**49**). Oleuropeinic acid (**31**) was present in specific cultivars in autumn (MAR, WAT, MAN, PIC, KOR) and was not detected in spring except for PIC. Nuezhenide (**14**) was known as the major phenolic compound in olive seeds^50^. It was detected previously in the leaves of the unique Australian olive cultivar Hardy’s Mammoth and certain Spanish cultivars^39^. Herein, nuezhenide was detected only in the leaf extracts of ASH and PIC in both seasons in addition to the two Greek cultivars (KOR, KAL) during spring.

To the best of our knowledge, a new compound namely, oleuropein-*O*-deoxyhexoside (**23**), was tentatively identified for the first time in nature. Oleuropein-*O*-deoxyhexoside (**23**); was detected only in the Spanish cultivar PIC during spring with marked decrease in oleuropein peak. Moreover, ethyl gallate (**6**) was detected only in ABQ during spring and its activity in the protection against diabetes has been previously reported^43^. In contrast to possible expectations, oleuropein was not the major compound detected in all the studied cultivars. Luteolin-*O*-hexoside was shown to be more predominant in some extracts. It was the main compound in HMD, WAT, ABQ, KOR during autumn; and TFH, PIC during spring.

## Conclusion

This work is considered the first comprehensive study for non-targeted metabolic profiling of olive leaves from different cultivars cultivated in Egypt. The study of different cultivars reveals the effect of both seasonal variation and genotype on the chemical composition of the leaves. HPLC–MS metabolic profiling of each extract can predict which family or compounds are available in a specific cultivar during the sampling time. In spring almost all the studied cultivars are characterized by high oleuropein, and various phenolic compounds, while in autumn the leaf extracts can serve as a rich source for bioactive pentacyclic triterpenes. Multivariate data analysis for HPLC–MS data showed that flavonoids and secoiridoids are the main contributors for cultivars segregation in both seasons. In autumn; secologanoside, oleoside, oleuropein-*O*-hexoside; oleuropein, oleuropein aglycone, luteolin-*O*-hexoside and diosmetin are the metabolites responsible for cultivar segregation. The Spanish cultivars were shown to be rich in secoirdoids, moreover, oleuropein was abundant in ASH and AOK, the Egyptian cultivars. PCA analysis of the ESI^−^ mode of autumn extracts, create a model that can discriminate between cultivars based on their botanical origin. In the future, the construction of cross-validated for further studies will be a valuable addition to the tool set of the robust PCR model structure. Finally, our data shed the light on the importance of olive leaves from Egyptian cultivars as an available low-cost byproduct for their utilization as a source of biologically active compounds.

## Materials and methods

### Plant material

Olive leaves of twelve different cultivars were collected in two different seasons: autumn (November 2015; during the full fruit maturation) and spring (April 2016; during the flowering stage) from the Horticulture Research Institute (HRI) in Giza, Egypt. Collection of plant material was carried out after permission from Prof. Dr. Mohamed El-Sayed, the chief researcher in Olive and Semi-arid Zone Fruits Research Department, Horticulture Research Institute. The collection and authentication of plant material occurred under his supervision. The collection complied with the IUCN Policy Statement on Research Involving Species at Risk of Extinction and collection requirements were carefully followed in the conduct of this research to comply with institutional, national, and international guidelines and legislation. Voucher specimens were deposited in the herbarium of Pharmacognosy Department, Faculty of Pharmacy, Ain Shams University with code numbers PHG-P-OE (169–180). The cultivars included six Egyptian, three Spanish, two Greek and one Italian cultivar. For this study, a sample of each cultivar was collected from three different trees, (n = 3) was obtained. After air drying, the leaves were grounded in a rotor mill and the dried powders were stored at 4 °C protected from light and moisture. The code, name and origin of each cultivar are shown in Table 2.

**Table 2:** Name, code, and origin of studied *Olea europaea* cultivars.

Sample code	Samplename	Origin
AOK	AgiziOkasi	Egypt
ASH	AgiziShami	Egypt
HMD	Hamed	Egypt
MRK	Maraki	Egypt
TFH	Toffahi	Egypt
WAT	Watiken	Egypt
MAN	Manzanillo	Spain
PIC	Picual	Spain
ABQ	Arbequina	Spain
KAL	Kalamata	Greece
KOR	Koroneiki	Greece
COR	Coratina	Italy

### Chemicals and reagents

Absolute ethanol of HPLC grade was obtained from fisher scientific, UK; Acetonitrile, methanol and formic acid (LC–MS grade) were obtained were obtained from Sigma-Aldrich, Steinheim, Germany, milliQ water was used for HPLC analysis. All other chemicals were purchased from Sigma-Aldrich (Merck, USA).

### Extracts preparation for HPLC–MS analysis

Selection of the most appropriate extraction method was based on previous work, where the highest yield of phenolic compounds was obtained using aqueous alcoholic solution^51–53^. The dried powders obtained for each cultivar were percolated in 70% ethanol for one day then filtered and the process was repeated two times in three consecutive days. The obtained liquid extract for each cultivar was concentrated with rotary evaporator (Büchi, Switzerland) and completely dried using a lyophilizer (Christ, Alpha 1–2 LD Plus) to yield a dry powder for each extract. The obtained powders were re-suspended in methanol for LC/MS analysis.

### HPLC–PDA-ESI–MS/MS analysis

HPLC–MS analysis was performed on a Finnigan LCQ-Duo ion trap mass spectrometer with an electrospray ionization source (ESI) ThermoQuest; coupled to a Finnigan Surveyor HPLC system. A gradient of water and acetonitrile with 0.1% formic acid (ESI^+^) and without in case of (ESI^−^) from 2 to 100% acetonitrile in 60 min at 30 °C. The flow rate was 0.5 ml/min. The injection volume was about 20 µl. All samples were measured in the positive and negative mode. The MS was operated with a capillary voltage of 10 V, source temperature of 240 °C, and high purity nitrogen as a sheath and auxiliary gas at a flow rate of 80 and 40, respectively. The ions were detected in a mass range of 50–2000 m*/z*. Collision energy of 35% was used in MS/MS for fragmentation. Data acquisitions and analyses were executed by Xcalibur™ 2.0.7 software (Thermo Scientific). Olive leaf extracts were analyzed in both positive and negative ionization modes. Metabolites identification was based on comparing the retention time, UV/Vis and mass spectra of each eluted compound with those reported in literature and online databases^54–57^.

### HPLC–ESI–MS data processing and multivariate data analysis

The whole mass profile for each cultivar was processed using MZmine2 version 2.39, an open-source software that is used for visualization and analysis of mass spectrometry based molecular profile data^58^. Thermo raw files obtained from Xcalibur are converted to NetCDF and then imported to MZmine software. LC/MS data processing based on chromatogram building and deconvlusion to individual peaks then aligned using RANSAC aligner. The resultant aligned peak list was further processed for gap filling step then exported to Microsoft Excel software to construct a data matrix containing all the aligned peaks *m/z* with their retention time and peak areas. The excel data matrix was subjected to PCA using the unscramble software to detect possible discrimination between cultivars and also to determine the main components responsible for the discrimination. All variables were mean centered and scaled to Pareto variance.

## Supplementary Information

Supplementary Information.

## Data Availability

Data are available upon request from the first author, Eman M. Kabbash.
