# Supplementary material for: Comparative metabolic profiling of olive leaf extracts from twelve different cultivars collected in both fruiting and flowering seasons
Source: Sci Rep. 2023 Jan 12;13:612. doi: 10.1038/s41598-022-27119-5 (PMC9837098; doi:10.1038/s41598-022-27119-5)
Supplement: Supplementary file 1 — Supplementary Information. [file 41598_2022_27119_MOESM1_ESM.docx]

**Supplementary material**

**Comparative metabolic profiling of olive leaf extracts from twelve different cultivars collected in both fruiting and flowering seasons**

Eman M. Kabbash^1^, Zeinab T. Abdel-Shakour^1^, Sherweit H. El-Ahmady^2,*^, Michael Wink ^3*^, and Iriny M. Ayoub^2*^

^1^ Phytochemistry Department, National Organization for Drug Control and Research, Giza, Egypt

^2^ Department of Pharmacognosy, Faculty of Pharmacy, Ain Shams University, Cairo 11566, Egypt

^3^ Institute of Pharmacy and Molecular Biotechnology, Heidelberg University, INF 364, D-69120 Heidelberg, Germany

| 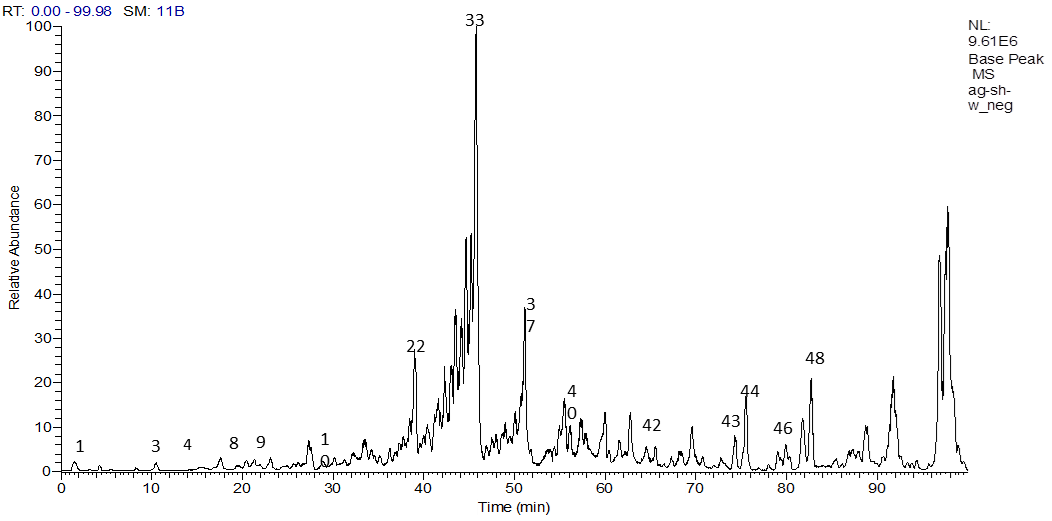  **A** | 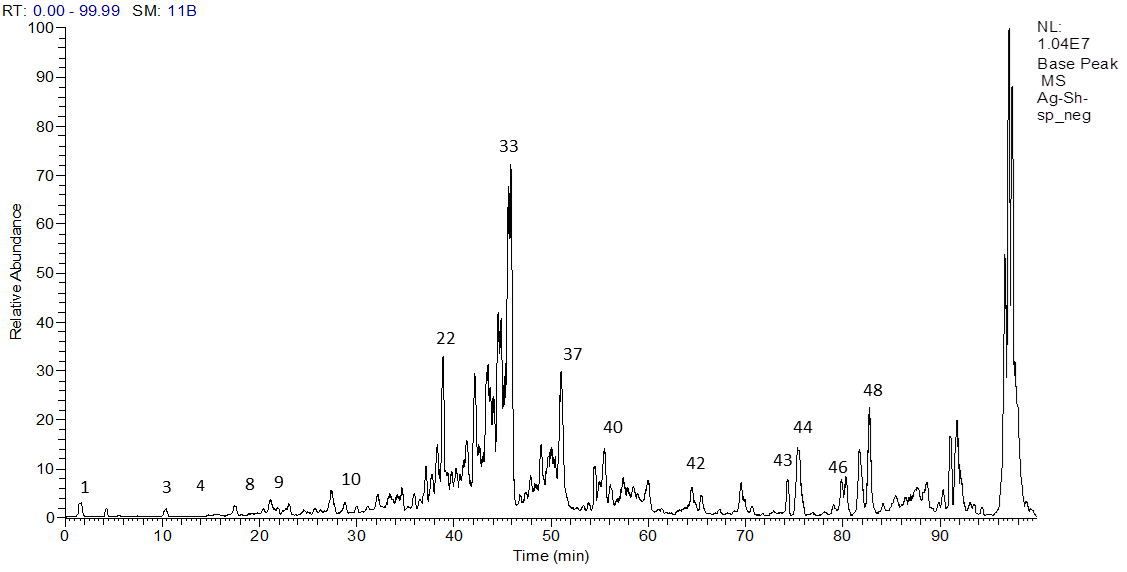  **B** |
| --- | --- |

| 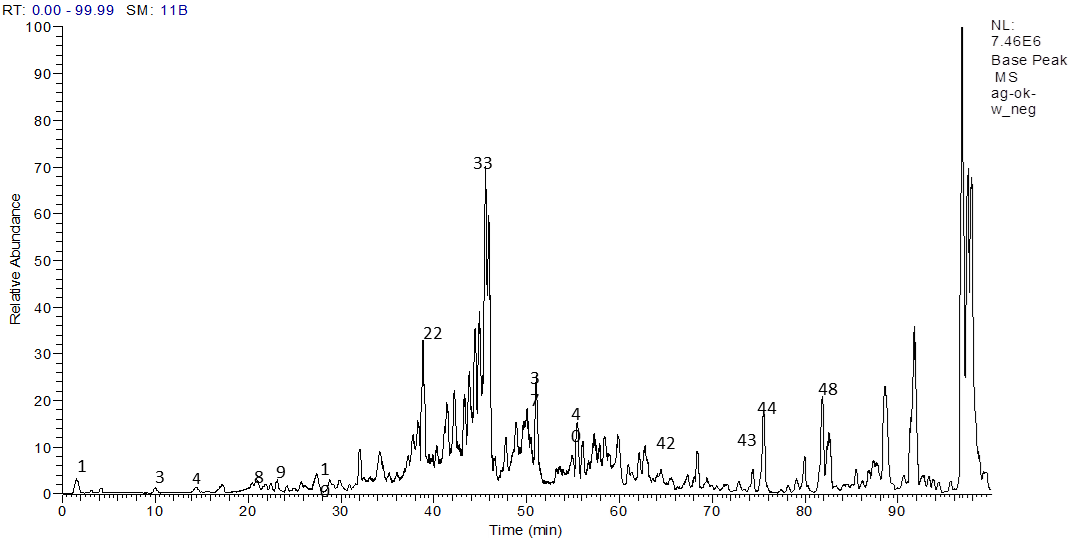  **C** | 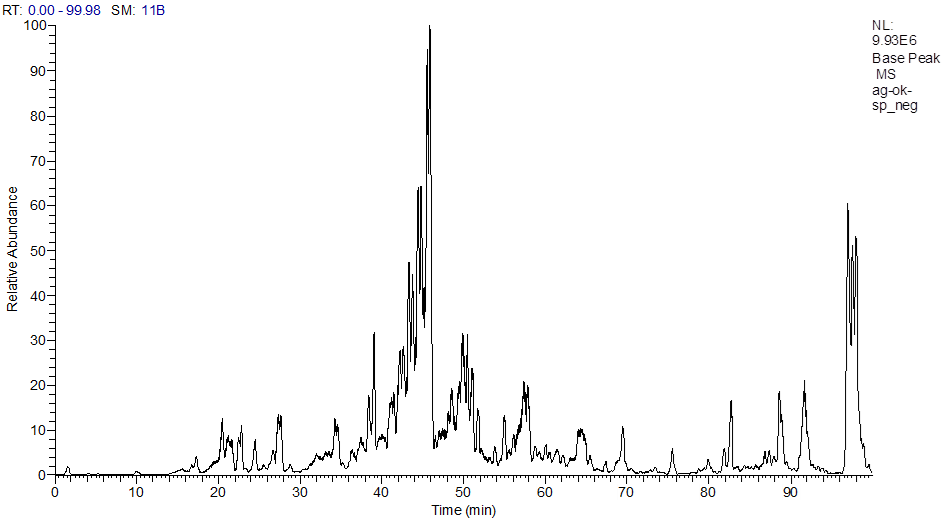  **D** |
| --- | --- |

| 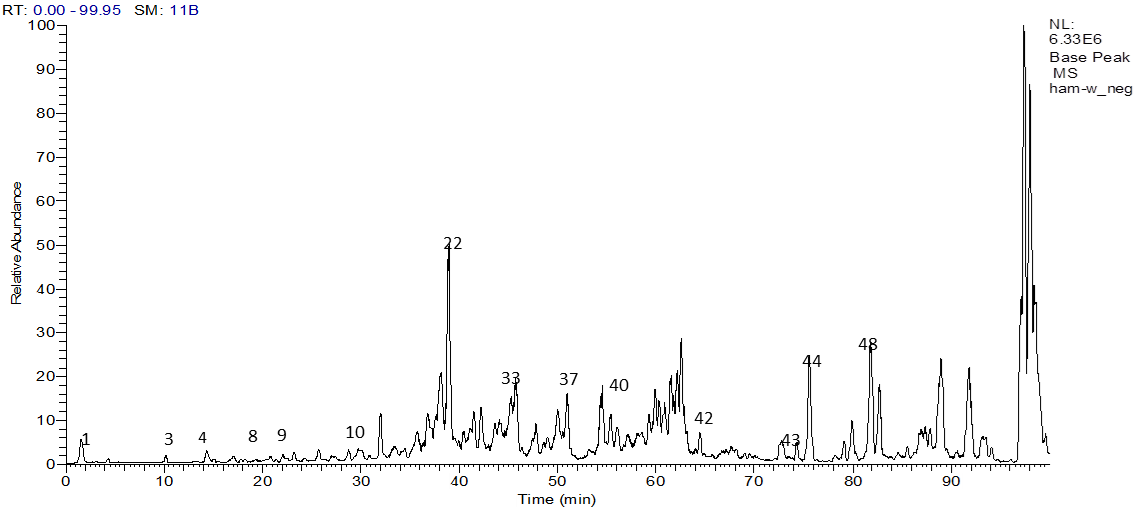  **E** | **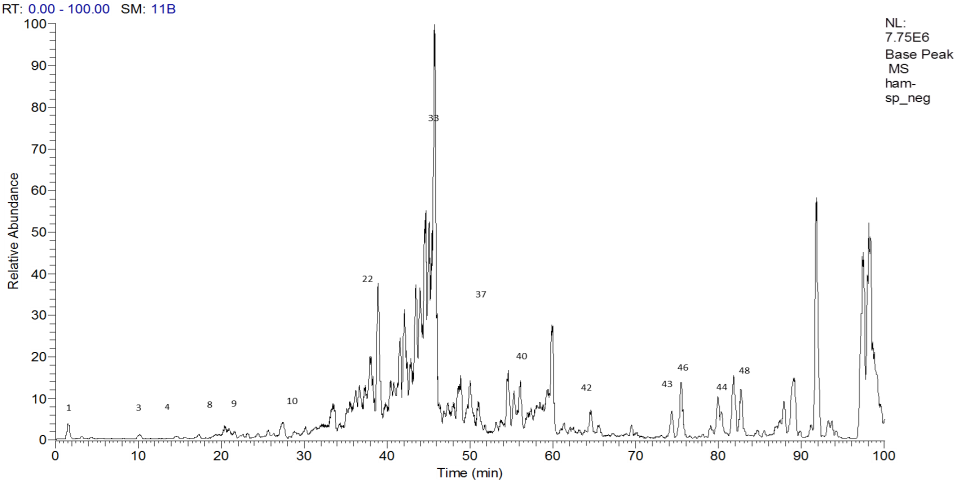**  **F** |
| --- | --- |

**Supplementary figure S1.** LC/MS base peak chromatograms of olive leaf extracts analysed in negative ionization mode ASH autumn extract (**A**), ASH spring extract (**B**), AOK autumn extract (**C**) and AOK spring extract (**D**), HMD autumn extract (**E**), HMD spring extract (**F**)

| 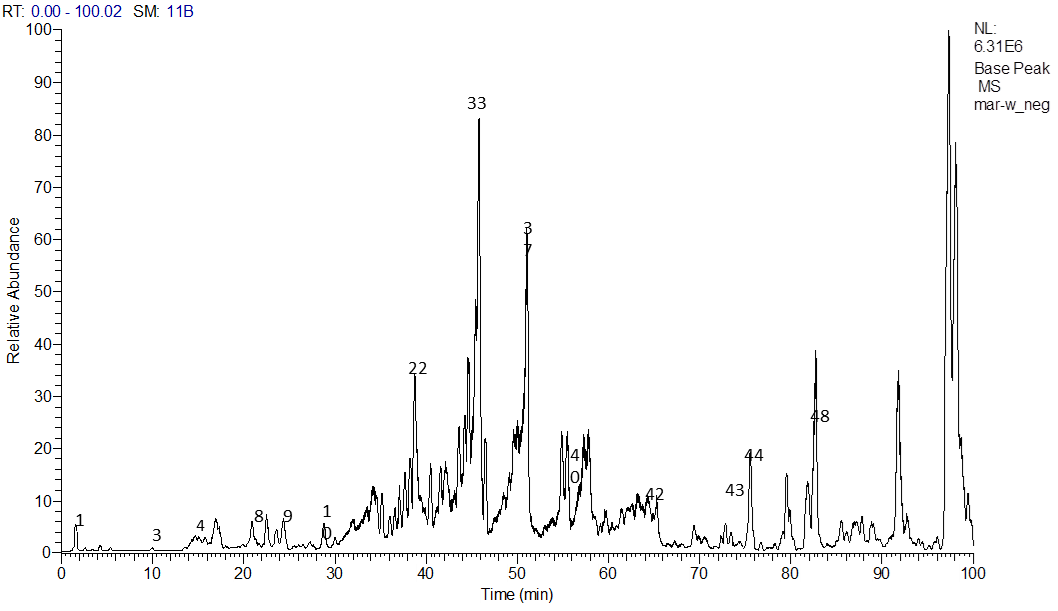  **G** | 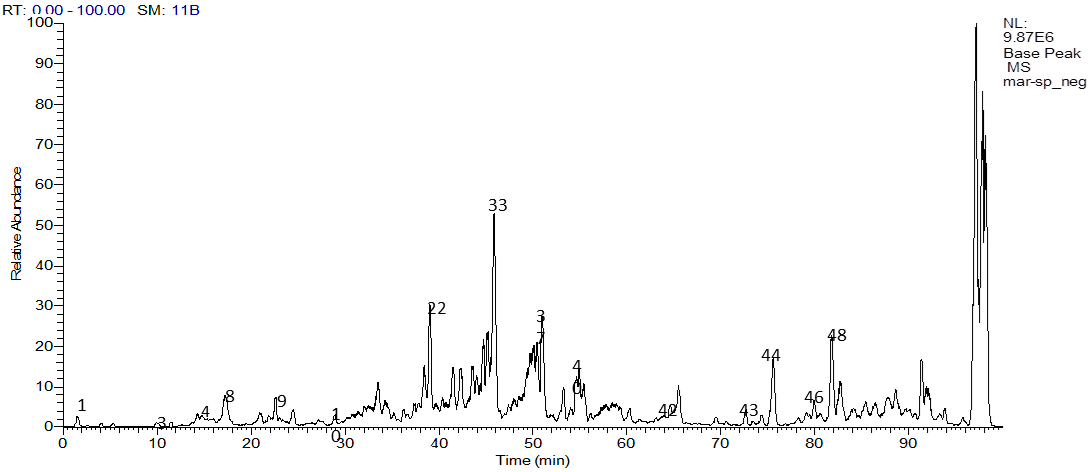  **H** |
| --- | --- |

| 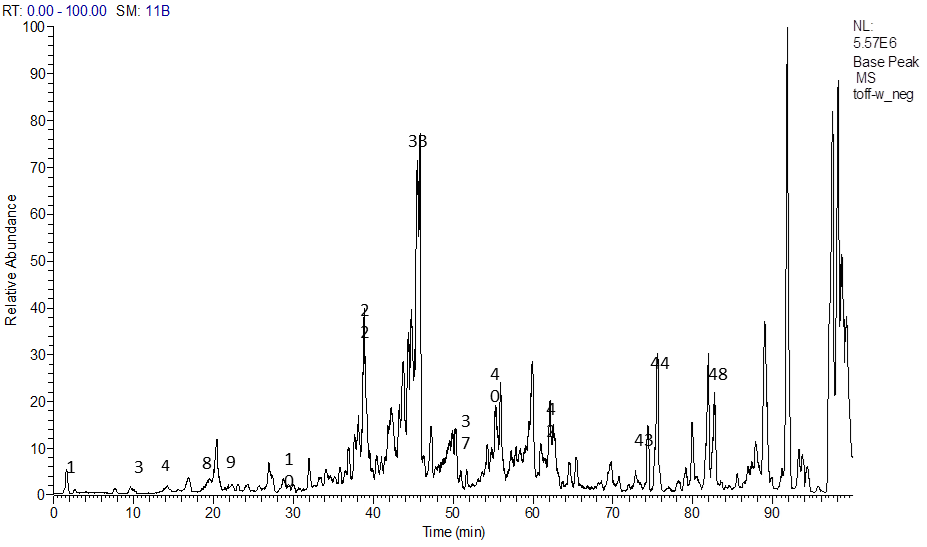  **I** | 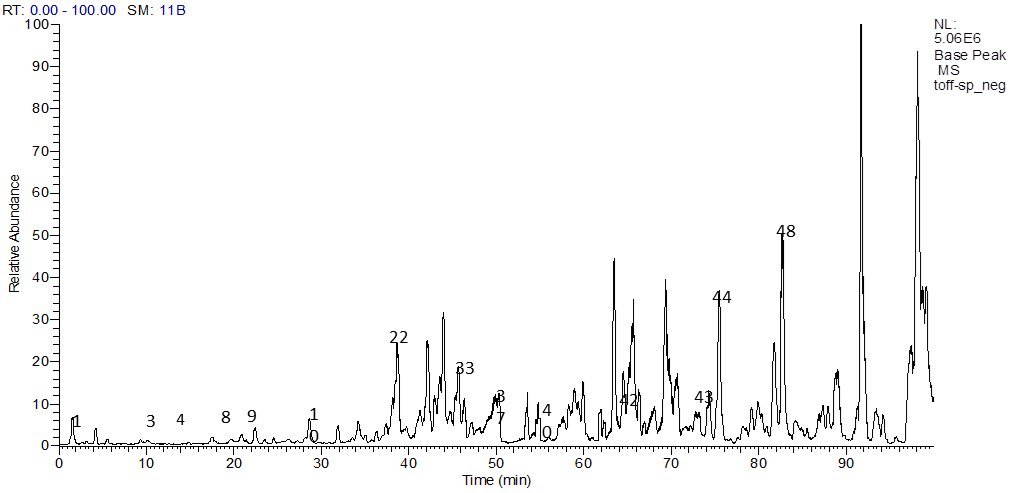  **J** |
| --- | --- |

| 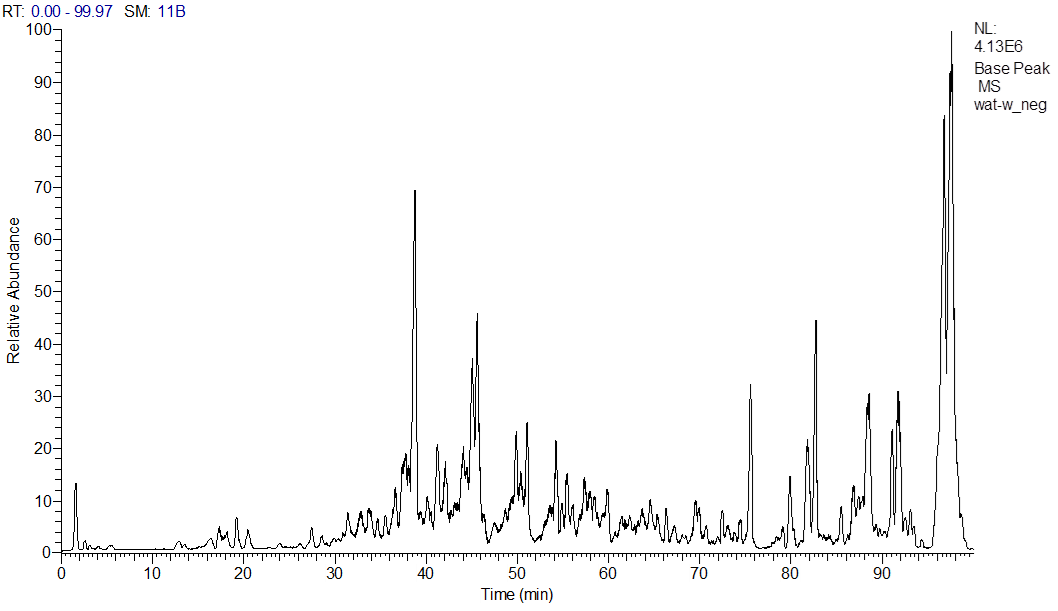  **K** | 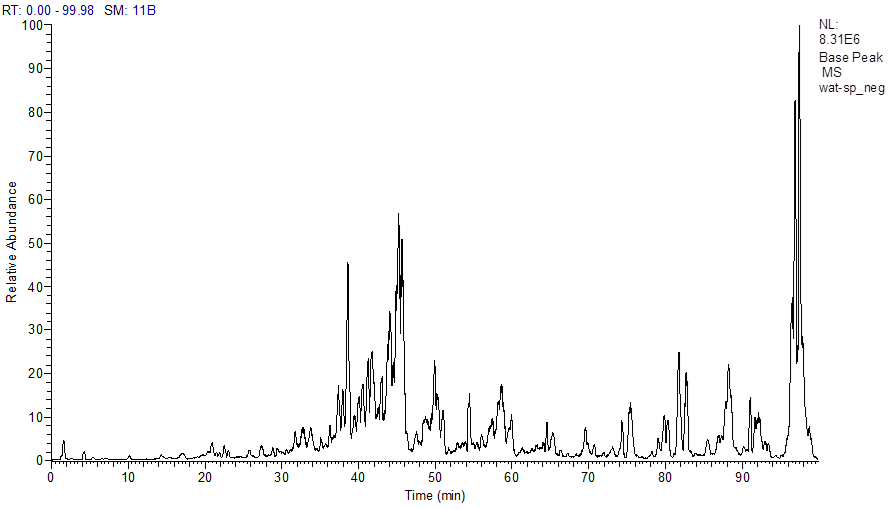  **L** |
| --- | --- |

**Supplementary figure S1. (Cont.)** LC/MS base peak chromatograms of olive leaf extracts analyzed in negative ionization mode MRK autumn extract (**G**), MRK spring extract (**H**), TFH autumn extract (**I**), TFH spring extract (**J**), WAT autumn extract (**K**), WAT spring extract (**L**)

| 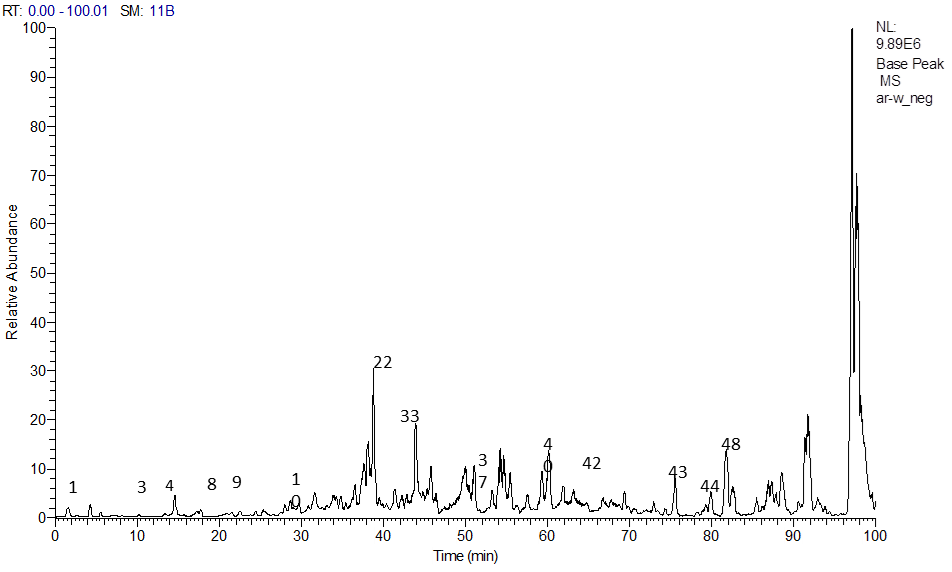  **M** | 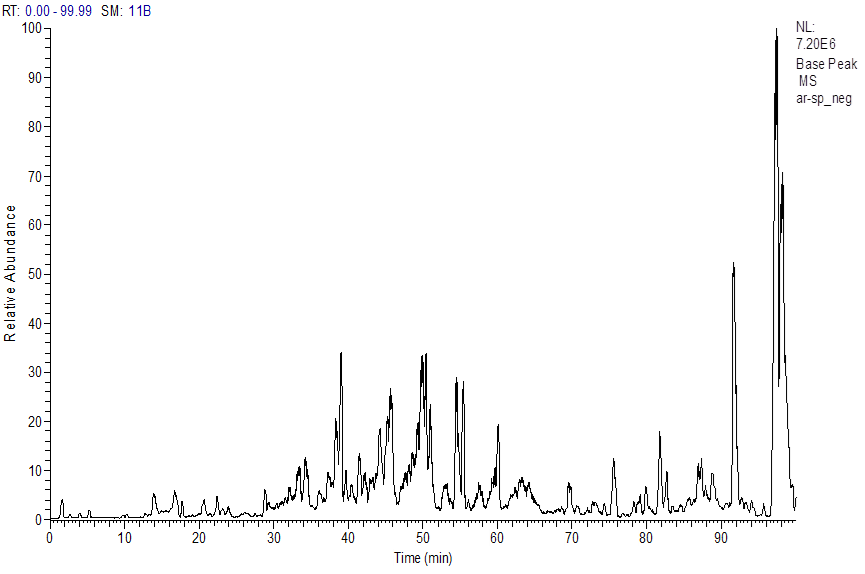  **N** |
| --- | --- |

| 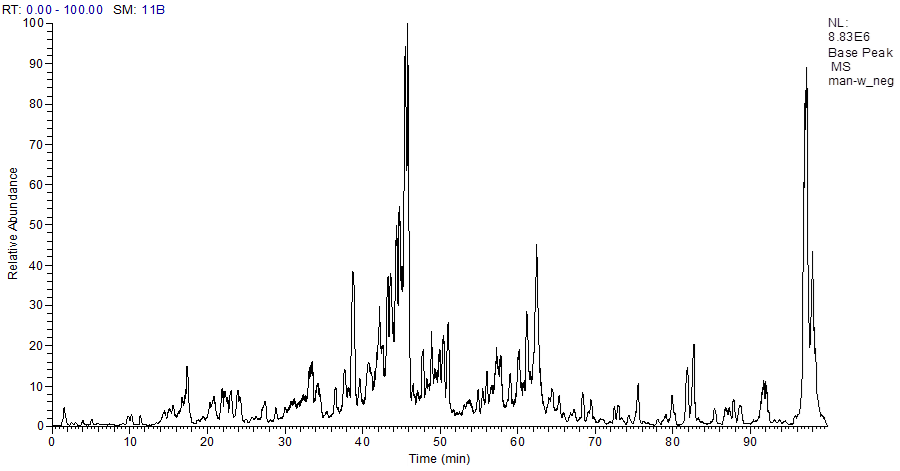  **O** | 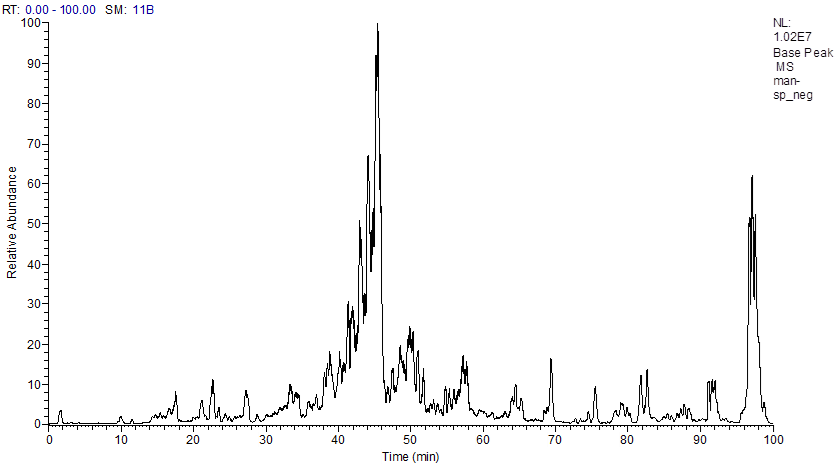  **P** |
| --- | --- |

| 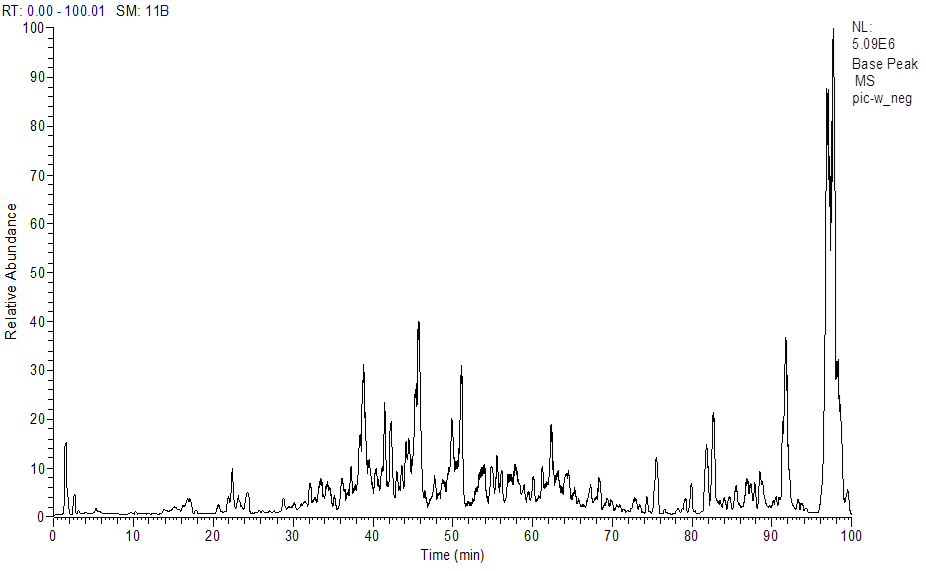  **Q** | 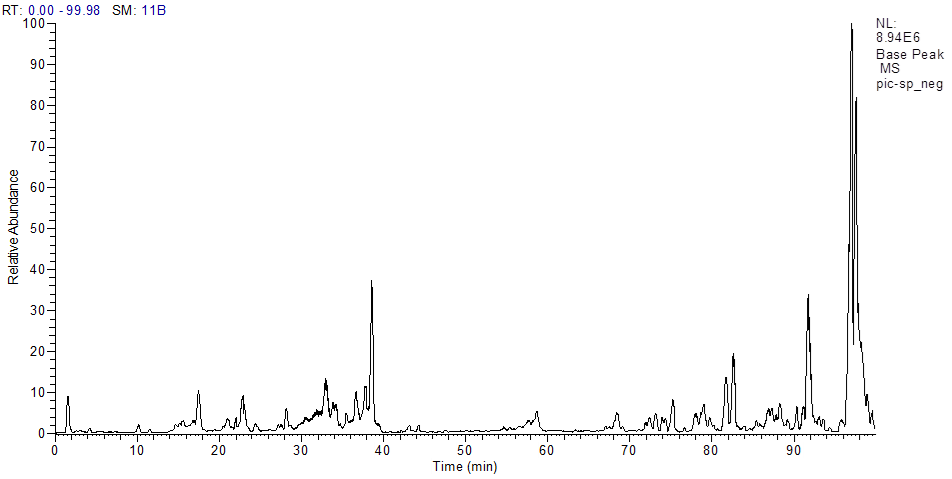  **R** |
| --- | --- |

**Supplementary figure S1. (Cont.)** LC/MS base peak chromatograms of olive leaf extracts analysed in negative ionization mode ABQ autumn extract (**M**), ABQ spring extract (**N**), MAN autumn extract (**O**), MAN spring extract (**P**), PIC autumn extract (**Q**), PIC spring extract (**R**)

| 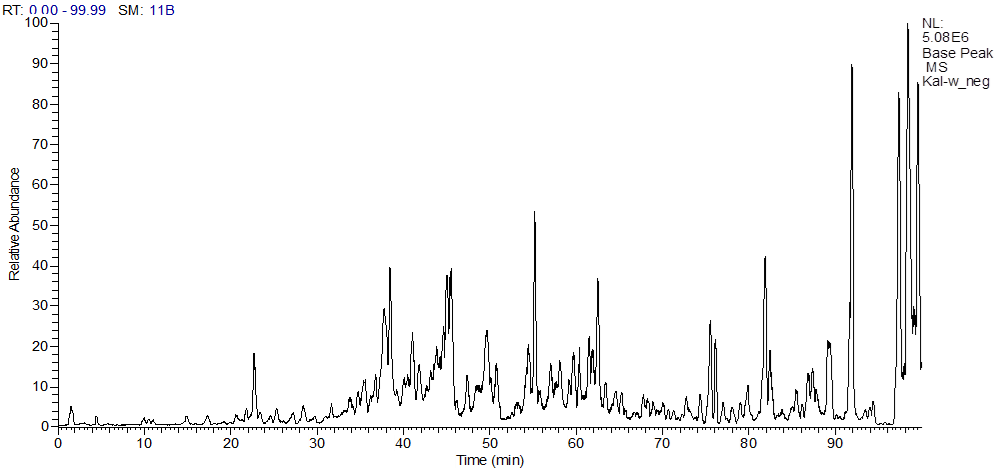  **T**  **S** | 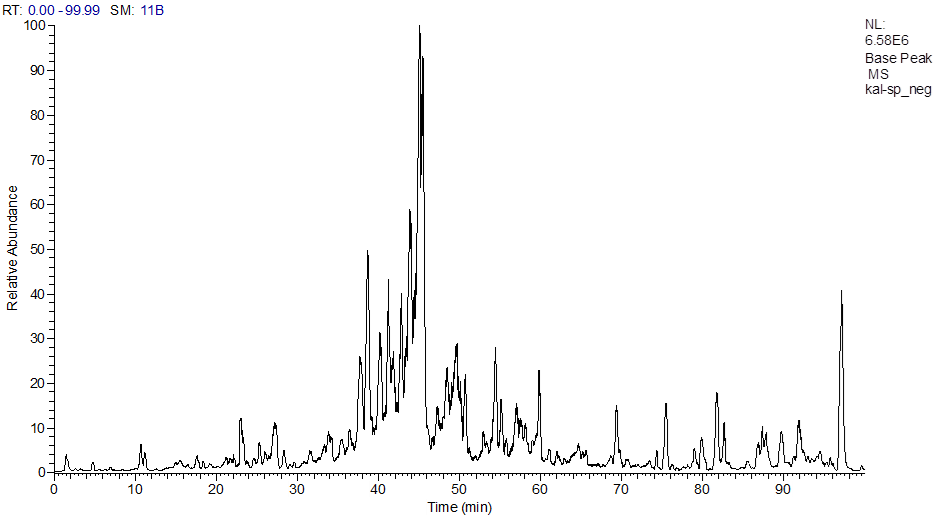 |
| --- | --- |

| 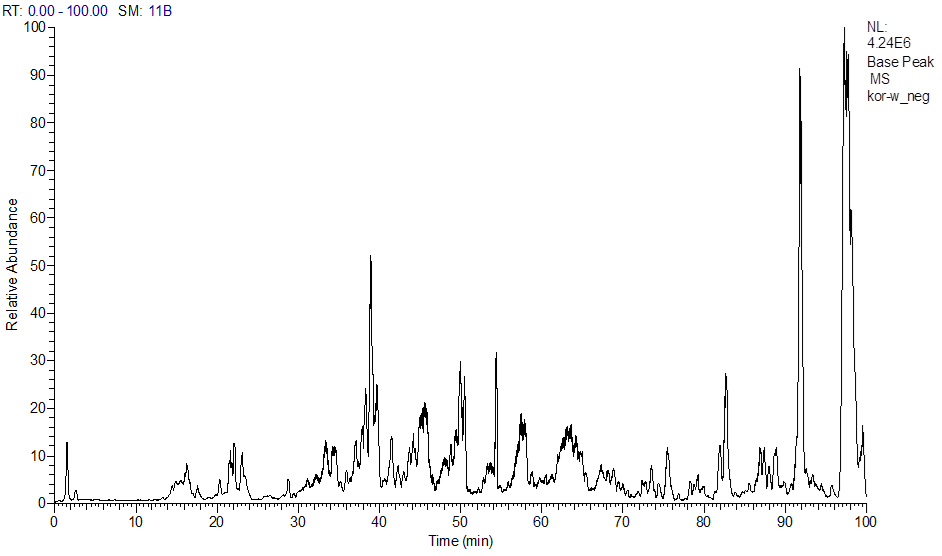  **V**  **U** | 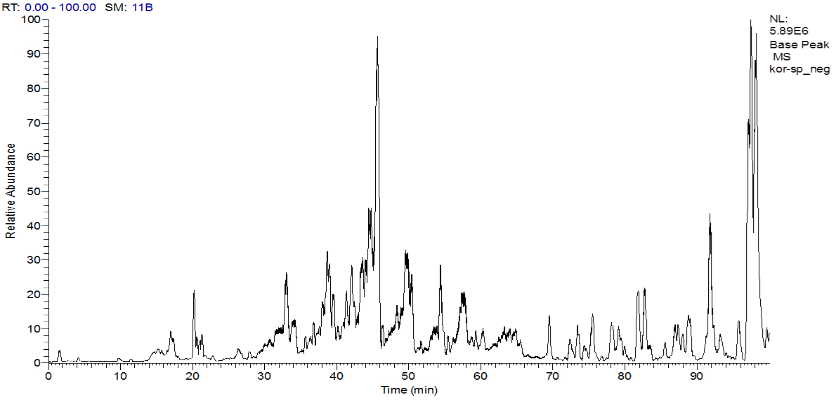 |
| --- | --- |

| 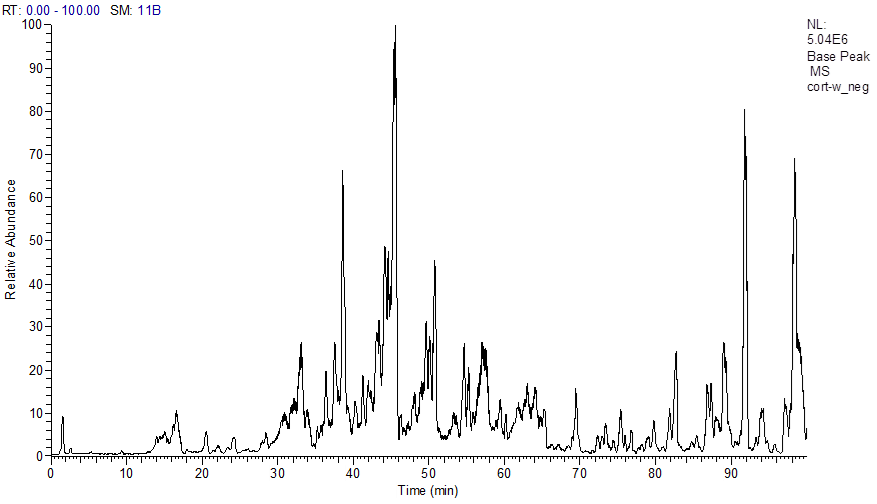  **X**  **W** | 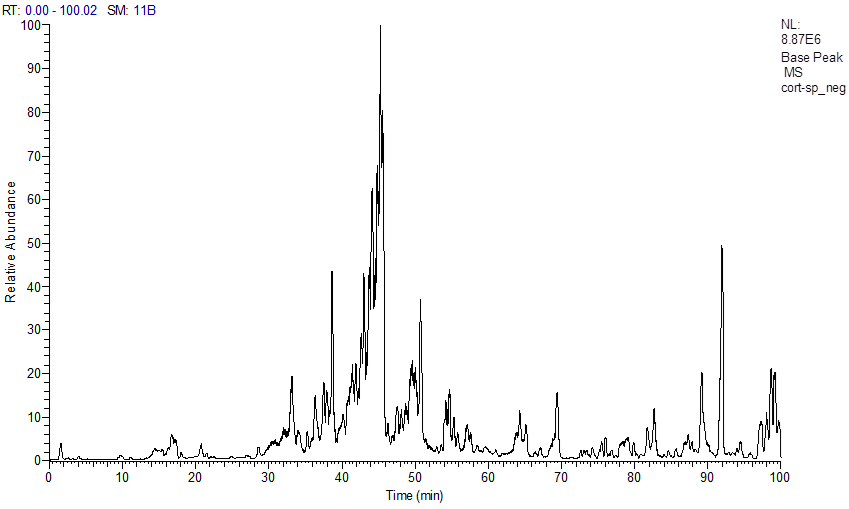 |
| --- | --- |

**Supplementary figure S1. (Cont.)** LC/MS base peak chromatograms of olive leaf extracts analyzed in negative ionization mode KAL autumn extract (**S**), KAL spring extract (**T**), KOR autumn extract (**U**), KOR spring extract (**V**), COR autumn extract (**W**), COR spring extract (**X**).


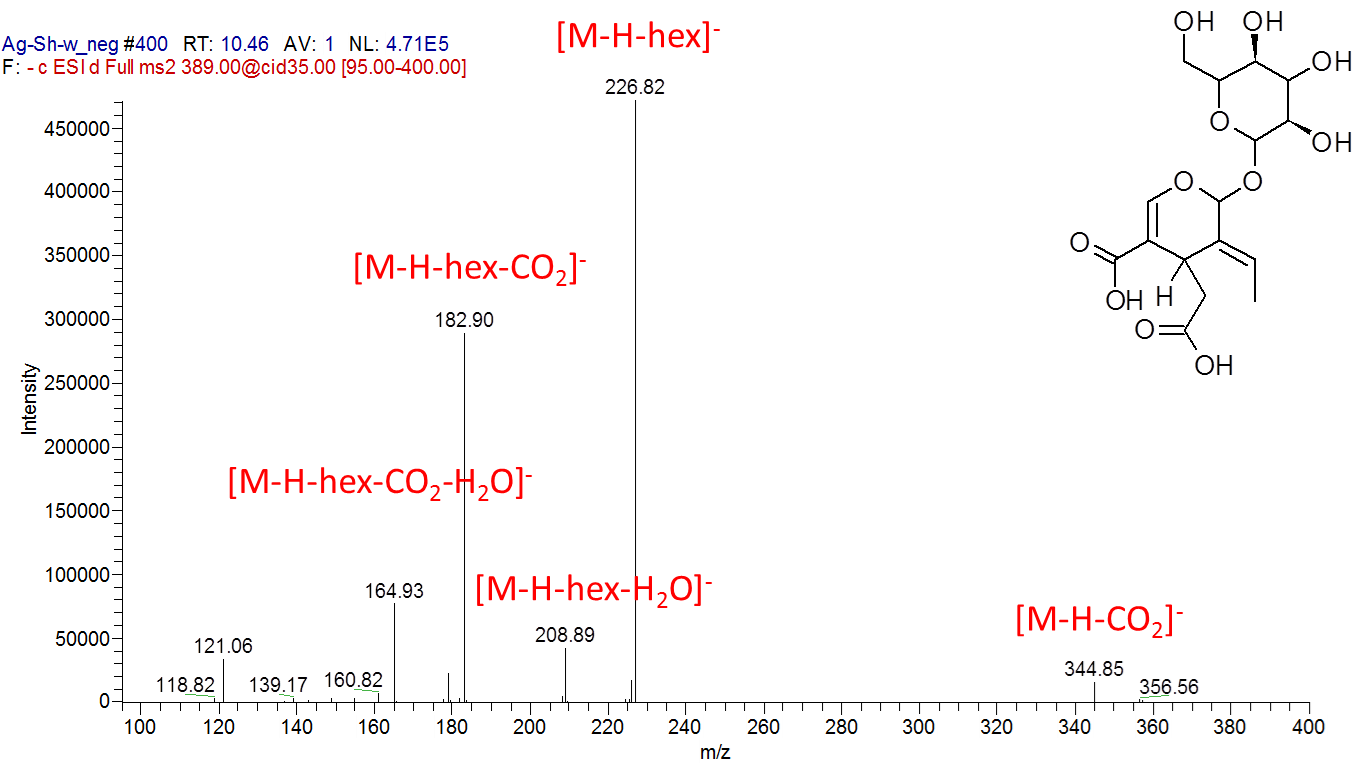


**Supplementary figure S2.** ESI-MS/MS spectrum of peak (**4**) in the negative ion mode showing oleoside at [M-H]^-^ 389.00


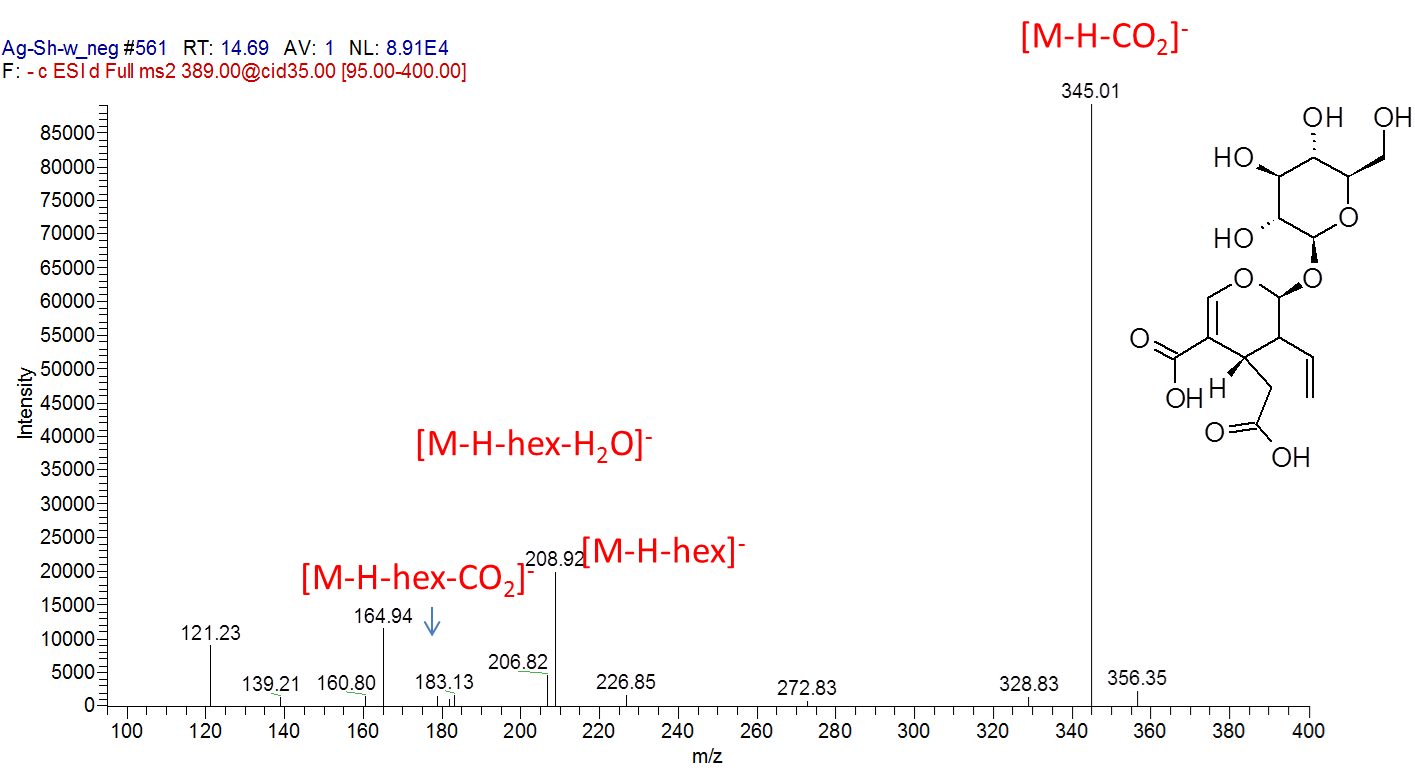


**Supplementary figure S3.** ESI-MS/MS spectrum of peak (5) in the negative ion mode showing secologanoside at [M-H]^-^ 389.00


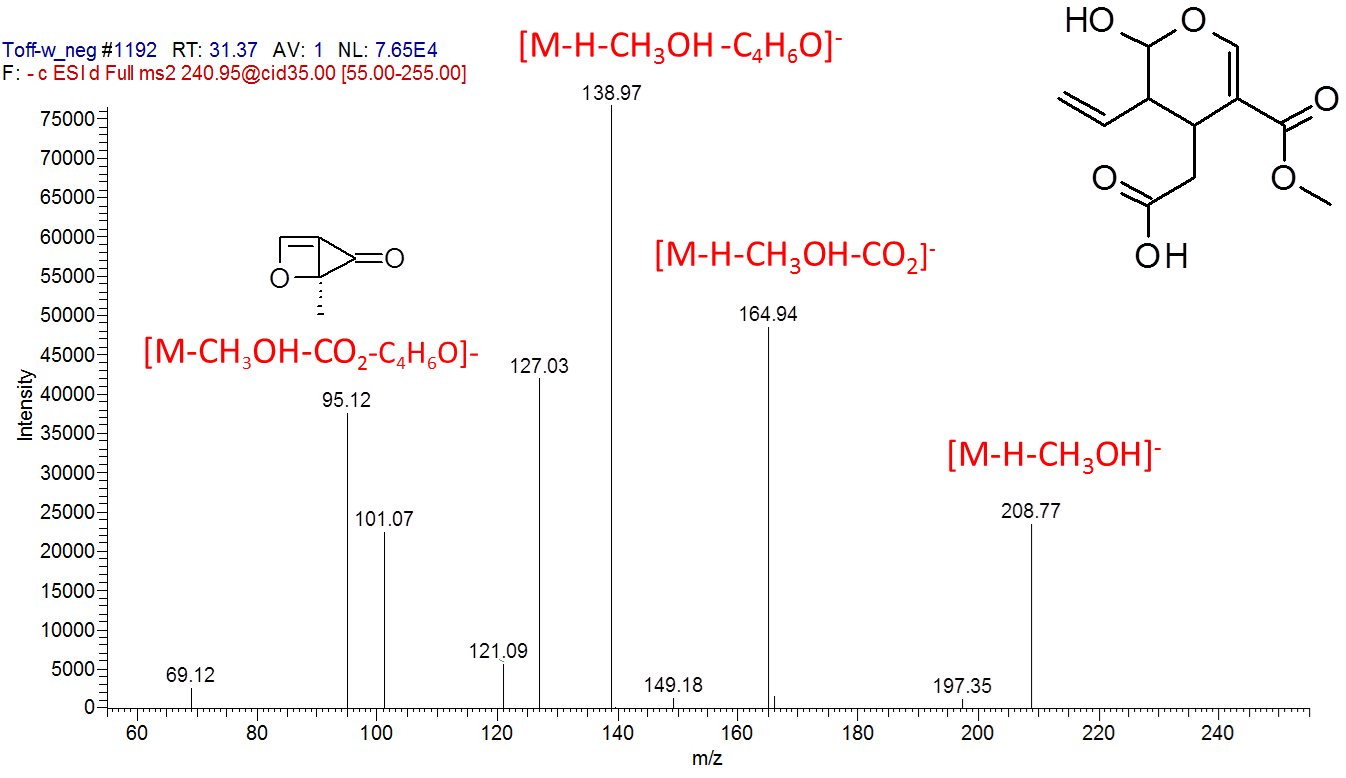
**Supplementary figure S4.** ESI-MS/MS spectrum of peak (10) in the negative ion mode showing elenolic acid at [M-H]^-^ 240.95


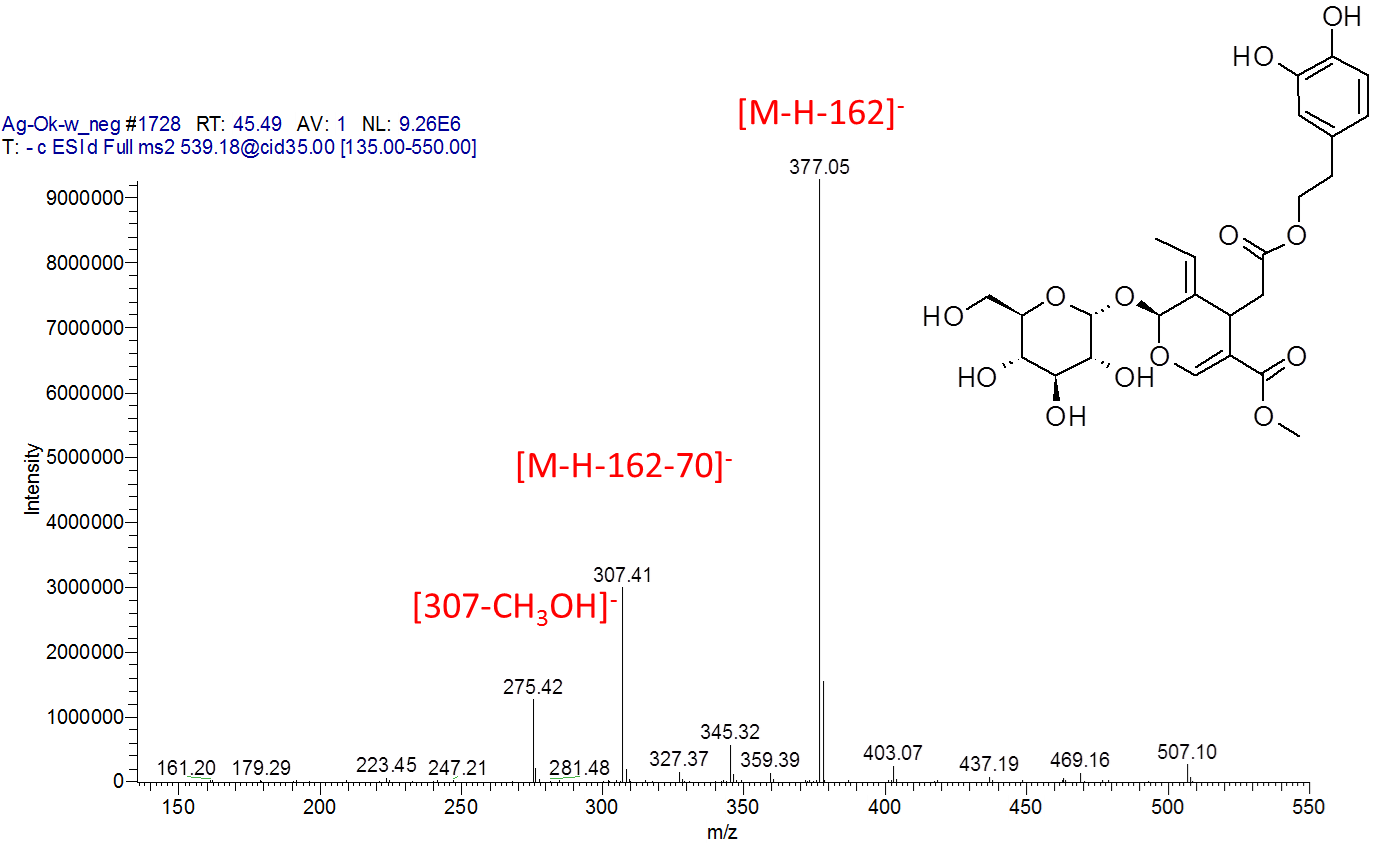
**Supplementary figure S5.** ESI-MS/MS spectrum of peak (33) in the negative ion mode showing oleuropein at [M-H]^-^ 539.18


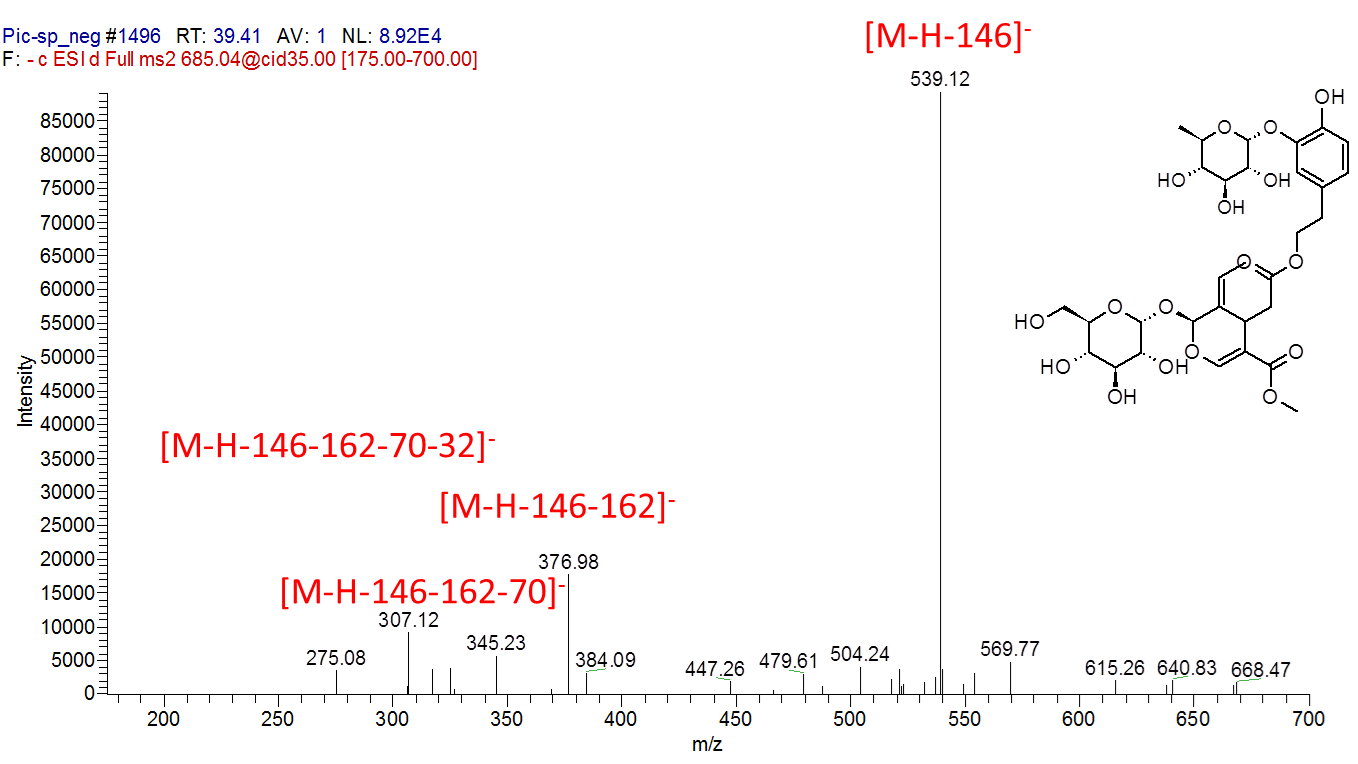
 **Supplementary figure S6.** ESI-MS/MS spectrum of oleuropein-*O*-deoxyhexoside (23) in the negative ion mode


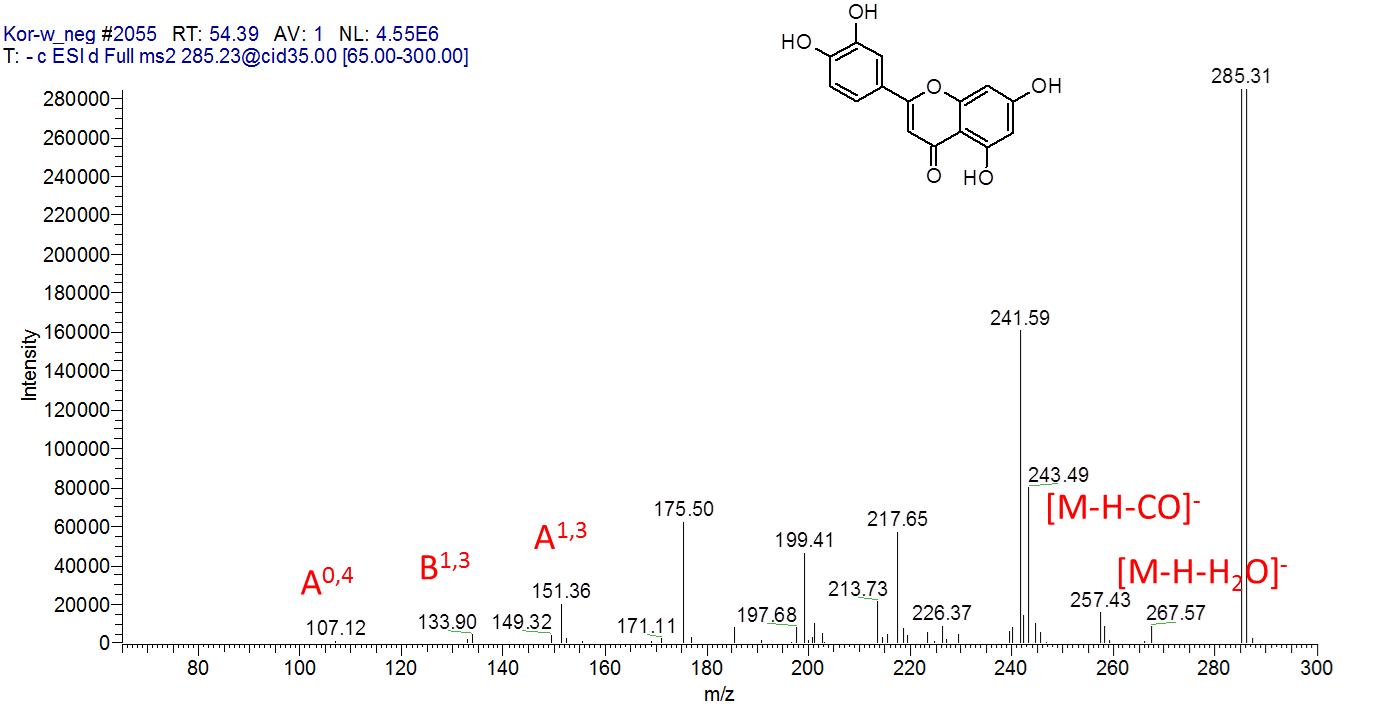
**Supplementary figure S7.** ESI-MS/MS spectrum of peak (**39**) in the negative ion mode showing **luteolin** at [M-H]^-^ 285.23
